# Dose Timing of D-Cycloserine to Augment Exposure Therapy for Social Anxiety Disorder: A Randomized Clinical Trial

**DOI:** 10.1001/jamanetworkopen.2020.6777

**Published:** 2020-06-04

**Authors:** Jasper A. J. Smits, Mark H. Pollack, David Rosenfield, Michael W. Otto, Sheila Dowd, Joseph Carpenter, Christina D. Dutcher, Elizabeth M. Lewis, Sara M. Witcraft, Santiago Papini, Joshua Curtiss, Leigh Andrews, Shelley Kind, Kristina Conroy, Stefan G. Hofmann

**Affiliations:** 1 Institute for Mental Health Research, Department of Psychology, The University of Texas at Austin, Austin; 2 Department of Psychiatry, Rush University Medical Center, Chicago, Illinois; 3 Now with Myriad Genetics, Salt Lake City, Utah; 4 Department of Psychology, Southern Methodist University, Dallas, Texas; 5 Department of Psychological and Brain Sciences, Boston University, Boston, Massachusetts; 6 Department of Psychology, Louisiana State University, Baton Rouge; 7 Department of Psychology, University of Mississippi, Oxford; 8 Department of Psychological and Brain Sciences, University of Delaware, Newark; 9 Department of Psychology, Suffolk University, Boston, Massachusetts; 10 Department of Psychology, Florida International University, Coral Gables

## Abstract

**Importance:**

Findings suggest that the efficacy of D-cycloserine (DCS) for enhancing exposure therapy may be strongest when administered after sessions marked by low fear at the conclusion of exposure practice. These findings have prompted investigation of DCS dosing tailored to results of exposure sessions.

**Objective:**

To compare tailored postsession DCS administration with presession DCS administration, postsession DCS administration, and placebo augmentation of exposure therapy for social anxiety disorder.

**Design, Setting, and Participants:**

This double-blind randomized clinical trial involved adults with social anxiety disorder enrolled at 3 US university centers. Symptom severity was assessed at baseline, weekly during treatment, and at 1-week and 3-month follow-up. Data analysis was performed from September 2019 to March 2020.

**Interventions:**

Participants completed a 5-session treatment and received pills commensurate with their condition assignment at sessions 2 through 5, which emphasized exposure practice.

**Main Outcomes and Measures:**

Symptom severity was evaluated by the Liebowitz Social Anxiety Scale and Social Phobic Disorders-Severity Form as administered by independent evaluators.

**Results:**

A total of 152 participants were enrolled (mean [SD] age, 29.24 [10.16] years; 84 men [55.26%]). Compared with placebo, presession and postsession conditions showed greater symptom improvement (*b* = −0.25; 95% CI, −0.37 to −0.13; *P* < .001; *d* = 1.07; and *b* = −0.20; 95% CI, −0.32 to −0.07; *P* = .002; *d* = 0.85) and lower symptom severity (*b* = −0.51; 95% CI, −0.81 to −0.21; *P* < .001; *d* = 0.76; and *b* = −0.49; 95% CI, −0.80 to −0.18; *P* = .002; *d* = 0.72) at 3-month follow-up. No differences were found between presession and postsession conditions. The tailored condition showed no advantage over placebo. Compared with the tailored condition, presession and postsession conditions evidenced greater decreases (*b* = −0.22; 95% CI, −0.34 to −0.10; *P* < .001; *d* = 0.94; and *b* = −0.17, 95% CI, −0.29 to −0.04; *P* = .008; *d* = 0.72) and lower symptom severity (*b* = −0.44, 95% CI, −0.73 to −0.14; *P* = .004; *d* = 0.64; and *b* = −0.41, 95% CI, −0.72 to −0.11; *P* = .008; *d* = 0.61) at 3-month follow-up.

**Conclusions and Relevance:**

Administration of DCS enhanced exposure therapy for social anxiety disorder when given before or after the exposure session. However, the study failed to achieve the aim to develop a tailored clinical application.

**Trial Registration:**

ClinicalTrials.gov Identifier: NCT02066792

## Key Points

**Question:**

What is the best dosing regimen for augmenting exposure therapy for social anxiety disorder with D-cycloserine?

**Findings:**

In this double-blind, placebo-controlled, randomized clinical trial involving 152 adults with social anxiety disorder, D-cycloserine augmented the treatment effects when administered before or after the exposure sessions. A tailored approach based on exposure success, as defined by low fear at the end of exposure practice, did not facilitate the therapy effects.

**Meaning:**

D-cycloserine augments exposure therapy for social anxiety disorder, although further optimizing the effects of D-cycloserine augmentation requires research on identifying the targets for tailored approaches.

## Teaser

This randomized clinical trial examines whether D-cycloserine administration augments exposure therapy for adults with social anxiety disorder.

## Introduction

In 2004, Ressler and colleagues^1^ published an article demonstrating that D-cycloserine (DCS), a partial agonist at the N-methyl-D-aspartate receptor, improved the efficacy of exposure therapy for height phobia. That study was a culmination of more than a decade of basic research associating the N-methyl-D-aspartate receptor with fear extinction consolidation—a mechanistic target for exposure therapy—and showing that DCS could effectively facilitate this learning process.^2^

The prospect of improving on a first-line intervention for anxiety disorders^3^ with a theoretically informed strategy generated considerable excitement.^4^ In the years that have followed, initial small-scale studies guided large-scale clinical trials of DCS efficacy across a number of fear-based disorders. In 2017, Mataix-Cols and colleagues^5^ summarized the findings of 21 studies in an individual participant data meta-analysis. Encompassing 5 disorders, with studies using different exposure therapy protocols and DCS applications (eg, dose, number of administrations, and timing of administration), the effect size for the advantage of DCS over placebo was statistically significant for pretreatment to posttreatment symptom improvement (*g* = 0.25), but not statistically significant for pretreatment to follow-up symptom improvement (*g* = 0.19).^5^ The effect size estimates were smaller than those reported in earlier meta-analyses,^6^ and in investigating this decline effect, Rosenfield and colleagues^7^ found that dosing parameters may be critical to DCS efficacy. Specifically, effect sizes were greater with more DCS administrations and when DCS was administered more than 60 minutes before the session. These associations accounted for the decline in effect sizes over time in the studies included in their meta-analysis.^7^

In addition to dosing characteristics, our group identified session success as a factor possibly associated with DCS efficacy. Across post hoc analyses of several studies, we observed that DCS augmentation was dependent on the level of fear patients reported at the end of the session, such that the advantage of DCS over placebo was stronger when end fear was lower vs when end fear was higher.^8,9^ We also observed this association for other cognitive enhancers (eg, yohimbine^10^ and methylene blue^11^), yet there have been failures to replicate this finding in other studies of DCS augmentation.^12,13^ Similarly, others have shown that session success defined by the degree of within-session fear reduction,^14^ between-session fear reduction,^15^ or threat reappraisal^16^ is associated with DCS efficacy.

Working toward developing a tailored DCS augmentation approach, the current study built upon these promising findings by testing whether judicious use—that is, DCS administration only after successful sessions—would optimize the efficacy of DCS augmentation.^17^ Informed by data from pilot work,^9,10^ we defined session success as achieving an end fear score of 40 or less on a scale of 0 to 100. We enrolled adults with social anxiety disorder (SAD) in a 5-session protocol, of which sessions 2 to 5 involved exposure and study medication. To test the efficacy of a tailored DCS administration strategy, we randomly assigned participants in a double-blind fashion to receive placebo before the session and either DCS after a successful session or placebo after an unsuccessful session (tailored), DCS before the session and placebo after the session (presession), placebo before the session and DCS after the session (postsession), or placebo before and after the session (placebo). We hypothesized that DCS augmentation would outperform placebo augmentation and that tailored DCS administration would yield greater outcomes compared with blanket DCS augmentation approaches.

## Methods

### Design

Before enrollment, participants provided written informed consent. The study was approved by the institutional review boards at the 3 sites (Boston University, Boston, Massachusetts; Rush University Medical Center, Chicago, Illinois; and The University of Texas at Austin), and a data and safety monitoring board provided oversight. This study follows the Consolidated Standards of Reporting Trials Extension (CONSORT Extension) reporting guideline.^18^

The study used a 3-site, double-blind, randomized design comparing tailored DCS administration, presession DCS administration, postsession DCS administration, and placebo administration for augmenting exposure therapy in adults with SAD. The exposure therapy protocol^17^ involved 1 psychoeducation session followed by 4 sessions focusing on exposure practice combined with pill administration. Randomization occurred at session 2, before the first instance of differential treatment.

The primary outcome, social anxiety symptom severity, was indexed by the Liebowitz Social Anxiety Scale (LSAS)^19^ and the Social Phobic Disorders-Severity Form (SPD-S)^20^ and was administered by evaluators blind to study condition at baseline, weekly during the intervention, and at 1-week, 1-month, and 3-month follow-up. We provide the trial protocol in Supplement 1 and have described the methods elsewhere.^17^

### Participants

Participants were adults who met the *Diagnostic and Statistical Manual of Mental Disorders* (Fifth Edition) (*DSM-5*) criteria for SAD and scored 60 or higher on the LSAS.^19^ Exclusion criteria were lifetime history of bipolar, psychotic, or obsessive-compulsive disorder; eating disorder, posttraumatic stress disorder, or substance use disorder in the past 6 months; any potentially interfering cognitive dysfunction; significant suicidal ideation or suicidal behaviors in the past 6 months; serious medical illness; history of seizures; pregnancy, lactation, or of childbearing potential and not using contraception; or concurrent psychotherapy or pharmacotherapy or prior nonresponse to exposure therapy.

### Procedures

#### Prerandomization

**Figure 1.**
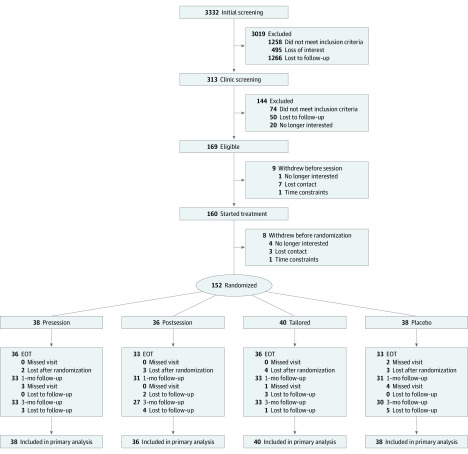
CONSORT Flow Diagram EOT indicates end of treatment (1-week follow-up).

Figure 1 depicts participant flow. Participants were recruited from February 15, 2015, to January 29, 2018. Potentially eligible participants who had completed an internet prescreen presented at the clinic (313 participants) to provide written informed consent and be evaluated for eligibility (eg, diagnostic clinical interview, administration of symptom severity measures, and medical evaluation). Race/ethnicity was assessed by self-report using options provided by the investigators as per the standard in previous trials. Those meeting eligibility criteria (169 participants) were also invited, before baseline assessment, to participate in an experiment designed to evaluate DCS efficacy for enhancing extinction of de novo threat conditioning (81 of 169 participated).^21^ At the baseline assessment, participants met with an independent evaluator who administered the outcome measures. The following week, 160 of 169 participants attended the first session of a 5-session group exposure therapy intervention.^17^

#### Postrandomization

The next week, before session 2, participants were assigned to a study condition (152 participants) (Figure 1). As overseen by one of the authors (D.R.), randomization occurred by site and by participation in the preexperiment, using variably sized permuted block randomization. Randomization tables were sent to the study pharmacists to prepare the medication kits. One hour before and immediately after sessions 2 through 5, participants were administered a pill commensurate with their condition assignment. Finally, independent evaluators administered outcome measures and assessed adverse events before each session and at the follow-up assessments. Participants received $25 for completing each follow-up assessment.

### Measures

As in previous protocols,^22,23^ trained staff administered the Structured Clinical Interview for *DSM-5*^24^ at Rush University and The University of Texas at Austin and the Anxiety Disorders Interview Schedule for *DSM-5*^25^ at Boston University. Similarly, trained evaluators blind to each participant’s condition administered the LSAS^19^ and the SPD-S.^20^ The LSAS is a 24-item scale that measures fear and avoidance in social and performance situations within the last week, using a scale of 0 (no fear or never avoids) to 3 (severe fear or usually avoids). The SPD-S is the Clinical Global Impression Scale^26^ adapted for SAD, which instructs evaluators to use a 7-point scale (1 denotes normal, not at all ill; 7 denotes among the most extremely ill patients) to index the severity of social anxiety.

During sessions 2 through 5, participants provided Subjective Units of Distress ratings (using the scale markers 0 = no fear, relaxed; 25 = mild fear, able to cope; 50 = moderate fear, trouble concentrating; 75 = severe fear, thoughts of leaving; and 100 = very severe fear, worst ever experienced)^27^ at the beginning of their exposure exercise (beginning fear) and just before the conclusion of their exposure exercise (end fear). They also reported the highest Subjective Units of Distress (peak fear) immediately after completing exposure practice. We used the end fear rating of 40 or less as an index of exposure success for determining which study pill was to be administered for the tailored condition.

### Treatment

#### Group Exposure Therapy

We used the 5-session group exposure therapy protocol^17^ used in previous studies.^10,16,22^ During the first session (60 minutes), clinicians provided education on SAD and a rationale for exposure therapy. Clinicians also oriented participants to the Subjective Units of Distress scale and participants practiced using the scale. Sessions 2 through 5 (90 minutes) focused on public speaking exposure practice. Each exposure exercise was planned with the participant to ensure adequate fear activation (ie, peak fear) and opportunity for violating harm expectancy.^28^ Clinicians encouraged participants to complete exposure practice between sessions. Groups typically consisted of 4 to 5 participants, but because of loss of participants during the prerandomization phase, a few group treatment cohorts involved only 1 or 2 participants. Therapy implementation procedures matched those of previous protocols.^10,22,23^ Specifically, groups were led by 2 clinicians (clinical psychologists or advanced doctoral students). Lead clinicians had completed a workshop and participated in at least 1 group as a colead clinician. Treatment integrity was ensured by weekly cross-site supervision led by one of the authors (J.A.J.S.) and an evaluation of 10% of randomly selected audio recorded sessions.

#### Study Medication

Study medication was administered and monitored by study staff blind to study condition. The DCS capsules contained 50 mg of DCS and polyethylene glycol 3350 powder, and the matching placebo capsules contained only polyethylene glycol 3350 powder.

### Statistical Analysis

Multilevel modeling (MLM) was used because it is recommended for analyzing longitudinal psychiatric data,^29^ accommodates missing data without requiring imputation, and includes all participants. Because there were 2 primary outcome variables (LSAS and SPD-S), we performed a multivariate MLM because it reduces type I error, increases power,^30^ and avoids inconsistent findings. The 4-level multivariate MLM analysis consisted of the 2 primary outcomes nested within repeated assessments over time, which were nested within individuals, which, in turn, were nested within treatment cohort. We compared various growth curve models and selected the logarithmic model because it demonstrated the lowest Akaike information criterion and Bayesian information criterion. Treatment group was coded using 3 dummy variables. To equalize groups on baseline severity and to account for otherwise random error, baseline LSAS and SPD-S were included as covariates. The outcome variables were *z*-scored.^30^ The significance threshold was set at 2-sided *P* < .05 for all significance tests. All data analyses were conducted using SPSS statistical software version 25.0 (IBM Corp). Effect sizes (Cohen *d*) were calculated as per Feingold.^31^ Finally, using the multilevel power analysis software PinT version 2.12 (Bosker RJ, Snijders TAB, Gulemond H) and assuming 30% missing data, a priori analysis indicated more than 0.80 power to detect condition differences of greater than *d* = 0.35 if the number of participants was greater than or equal to 148. Hence, targeted sample size was set slightly higher, at 156. Data analysis was performed from September 2019 to March 2020.

## Results

This clinical trial involved 152 adults with SAD (mean [SD] age, 29.24 [10.16] years; 84 men [55.26%]); 52 participants were at Boston University, 49 were at Rush University Medical Center, and 51 were at The University of Texas at Austin. There were no between-group differences on demographic variables or outcome measures at baseline (Table 1). For example, in the presession, postsession, tailored, and placebo groups, respectively, 22 (57.89%), 21 (58.33%), 19 (47.5%), and 22 (57.89%) participants were female; 23 (57.5%), 18 (45.0%), 25 (62.5%), and 24 (60.0%) participants were white; 27 (71.05%), 30 (83.33%), 27 (67.50%), and 27 (71.05%) participants were single; 28 (73.68%), 30 (83.33%), 29 (72.50%), and 29 (76.32%) participants lived in urban areas; 20 (52.63%), 15 (41.67%), 19 (47.50%), and 19 (50.0%) participants were employed full time; the mean (SD) baseline scores on the LSAS were 80.61 (14.73), 86.25 (18.43), 85.65 (15.05), and 85.24 (16.03); and the mean (SD) baseline scores on the SPD-S were 5.53 (0.65), 5.75 (0.77), 5.63 (0.67), and 5.63 (0.71).

**Table 1.** Sample Characteristics

Characteristic	Participants, No. (%)			
	Presession (n = 38)	Postsession (n = 36)	Tailored (n = 40)	Placebo (n = 38)
Age, mean (SD), y	29.73 (10.42)	27.54 (8.29)	30.73 (9.88)	28.76 (11.81)
Sex				
Male	16 (42.11)	15 (41.67)	20 (50.00)	16 (42.11)
Female	22 (57.89)	21 (58.33)	19 (47.50)	22 (57.89)
Genderqueer	0	0	1 (2.50)	0
Race				
White	23 (57.50)	18 (45.00)	25 (62.50)	24 (60.00)
Black or African American	5 (12.50)	3 (7.50)	8 (20.00)	4 (10.00)
Asian	7 (17.50)	13 (32.50)	6 (18.00)	8 (20.00)
Other	1 (2.50)	2 (5.00)	0	2 (5.00)
Not reported	2 (5.00)	0	1 (2.50)	0
Ethnicity				
Not Hispanic or Latino	32 (84.21)	25 (69.44)	33 (82.50)	26 (68.42)
Hispanic or Latino	6 (15.79)	9 (25.00)	5 (12.50)	10 (26.32)
Not reported	0	2 (5.56)	2 (5.00)	2 (5.26)
Marital status				
Single	27 (71.05)	30 (83.33)	27 (67.50)	27 (71.05)
Living with partner	3 (7.89)	2 (5.56)	4 (10.00)	6 (15.79)
Married	8 (21.05)	3 (8.33)	7 (17.50)	4 (10.53)
Divorced	0	1 (2.78)	2 (5.00)	1 (2.63)
Highest education				
Graduate school	14 (36.84)	13 (36.11)	18 (45.00)	5 (13.16)
College graduate	14 (36.84)	9 (25.00)	12 (30.00)	17 (44.74)
Partial college	8 (21.05)	13 (36.11)	9 (22.50)	14 (36.84)
High school graduate	1 (2.63)	1 (2.78)	1 (2.50)	2 (5.26)
Partial high school	1 (2.63)	0	0	0
Highest occupation				
Executive	0	1 (2.78)	1 (2.50)	0
Manager or professional	15 (39.47)	13 (36.11)	15 (37.50)	9 (23.68)
Administrative	5 (13.16)	5 (13.89)	5 (12.50)	7 (18.42)
Clerical	3 (7.89)	1 (2.78)	1 (2.50)	7 (18.42)
Skilled	9 (23.68)	5 (13.89)	7 (17.50)	7 (18.42)
Semiskilled	3 (7.89)	4 (11.11)	8 (20.00)	6 (15.79)
Unskilled	2 (5.26)	4 (11.11)	1 (2.500)	0
Never worked	1 (2.63)	3 (8.33)	2 (5.00)	2 (5.26)
Living situation				
Urban	28 (73.68)	30 (83.33)	29 (72.50)	29 (76.32)
Suburban	8 (21.05)	6 (16.67)	11 (27.50)	8 (21.05)
Rural	2 (5.26)	0	0	1 (2.63)
Annual income, $				
Not given	8 (21.05)	4 (11.11)	7 (17.50)	9 (23.68)
0-4999	3 (7.89)	10 (27.78)	4 (10.00)	4 (10.53)
5000-9999	2 (5.26)	1 (2.78)	2 (5.00)	1 (2.63)
10 000-14 999	2 (5.26)	2 (5.56)	1 (2.50)	4 (10.53)
15 000-24 999	2 (5.26)	3 (8.33)	5 (12.50)	5 (13.16)
25 000-34 999	5 (13.16)	4 (11.11)	2 (5.00)	3 (7.89)
35 000-49 999	5 (13.16)	0 (0)	6 (15.00)	6 (15.79)
50 000-74 999	9 (23.68)	6 (16.67)	7 (17.50)	2 (5.26)
≥75 000	2 (5.26)	6 (16.67)	6 (15.00)	4 (10.53)
Occupational status				
Not applicable	2 (5.26)	5 (13.89)	3 (7.50)	7 (18.42)
Full-time	20 (52.63)	15 (41.67)	19 (47.50)	19 (50.00)
Part-time	7 (18.42)	6 (16.67)	6 (15.00)	5 (13.16)
Dependent on spouse or is a student	9 (23.68)	10 (27.78)	12 (30.00)	7 (18.42)
Baseline scores, mean (SD)				
Liebowitz Social Anxiety Scale	80.61 (14.73)	86.25 (18.43)	85.65 (15.05)	85.24 (16.03)
Social Phobic Disorders-Severity Form	5.53 (0.65)	5.75 (0.77)	5.63 (0.67)	5.63 (0.71)

Session attendance was high (139 participants [91.4%] attended session 5) and attrition rates were low (29 participants [19.1%]) and these did not differ between groups. Also, participants’ demographic and baseline measures were not different between those with missing vs those with complete data. Adverse effects potentially attributable to the drug were mild, consistent with the known profile of DCS.^32^ Finally, χ^2^ analyses revealed no between-group differences with respect to participants’ guesses concerning pill ingredients (DCS vs placebo).

Because participants’ end fear determined pill administration (DCS vs placebo) in the tailored condition, we tested whether end, beginning, or peak fear across the sessions varied among conditions. Analyses of variance performed using MLM showed no interactions between conditions and sessions and no condition differences on these indices (Table 2). For example, the mean (SD) end fear scores for the presession, postsession, tailored, and placebo groups, respectively, were 55.66 (19.53), 49.31 (22.08), 49.55 (18.83), and 53.95 (20.47) for session 2; 48.97 (18.22), 42.26 (19.15), 42.95 (14.58), and 47.77 (16.49) for session 3; 50.00 (19.06), 46.12 (21.29), 45.32 (13.91), and 49.77 (16.78), for session 4; and 45.56 (14.53), 41.35 (15.07), 44.57 (16.60), and 44.31 (13.92) for session 5. Similarly, there were no condition effects in a generalized linear mixed model for the proportion of sessions meeting the success criterion (Table 2). Hence, fear levels were comparable across experimental conditions. Finally, the mean (SD) number of DCS doses received by participants in the tailored condition was 2.18 (1.53), which was statistically significantly lower than the number received by those in presession (mean [SD], 3.92 [0.27] doses) and postsession (mean [SD], 3.78 [0.76] doses) conditions (*F*_2,111_ = 35.47; *P* < .001).

**Table 2.** Fear Ratings and Session Success

Fear ratings	Score, mean (SD)			
	Presession (n = 38)	Postsession (n = 36)	Tailored (n = 40)	Placebo (n = 38)
Session 2				
Beginning	68.45 (16.41)	70.28 (15.30)	68.82 (15.19)	68.16 (15.13)
Peak	79.74 (12.84)	79.31 (14.35)	77.05 (14.14)	80.59 (14.47)
End	55.66 (19.53)	49.31 (22.08)	49.55 (18.83)	53.95 (20.47)
Success rate, %	28.95	47.22	47.50	34.21
Session 3				
Beginning	63.69 (12.00)	63.86 (14.35)	66.5 (14.64)	63.43 (14.64)
Peak	74.51 (10.96)	72.40 (14.32)	76.05 (11.71)	71.71 (13.23)
End	48.97 (18.22)	42.26 (19.15)	42.95 (14.58)	47.77 (16.49)
Success rate, %	50.00	64.71	67.50	45.71
Session 4				
Beginning	66.51 (13.90)	66.24 (15.12)	68.05 (14.78)	65.8 (17.46)
Peak	76.51 (15.57)	72.88 (14.0 6)	77.51 (9.88)	74.34 (16.83)
End	50.00 (19.06)	46.12 (21.29)	45.32 (13.91)	49.77 (16.78)
Success rate, %	42.11	45.45	51.35	42.86
Session 5				
Beginning	60.83 (13.76)	64.12 (13.00)	63.86 (14.81)	65.31 (14.25)
Peak	71.47 (13.80)	72.94 (11.49)	71.20 (13.04)	73.22 (13.27)
End	45.56 (14.53)	41.35 (15.07)	44.57 (16.60)	44.31 (13.92)
Success rate, %	57.14	57.58	62.86	62.50

**Figure 2.**
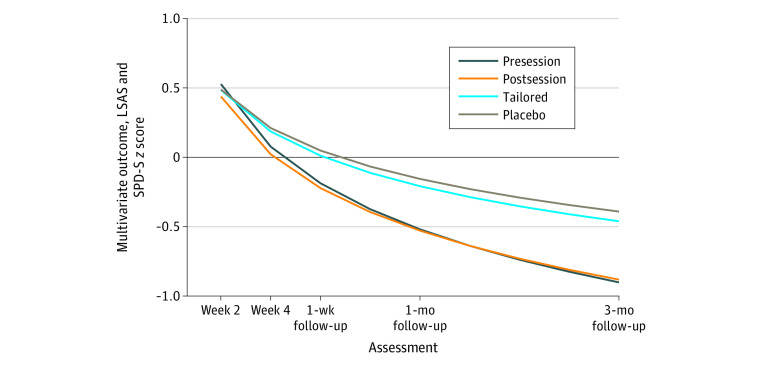
Multivariate Outcome by Treatment Condition at Each Assessment Point LSAS indicates Liebowitz Social Anxiety Scale; and SPD-S, Social Phobic Disorders-Severity Form (SPD-S).

Participants in the presession and postsession conditions improved faster than did participants in the placebo condition (*b* = −0.25; 95% CI, −0.37 to −0.13; *t*_815_ = −4.05; *P* < .001; *d* = 1.07; and *b* = −0.20; 95% CI, −0.33 to −0.07; *t*_822_ = −3.07; *P* = .002; *d* = 0.85) and had lower severity at 3-month follow-up (*b* = −0.51; 95% CI, −0.81 to −0.21; *t*_261_ = −3.34; *P* < .001; *d* = 0.76; and *b* = −0.49; 95% CI, −0.80 to −0.18; *t*_279_ = 3.08; *P* = .002; *d* = 0.72) (Figure 2 and Table 3). For example, at week 18, for the presession, postsession, tailored, and placebo groups, respectively, the raw mean (SD) LSAS scores were 47.58 (22.37), 49.44 (25.58), 59.64 (24.32), and 65.07 (27.96); and the raw mean (SD) SPD-S scores were 3.21 (1.70), 3.50 (1.75), 4.12 (1.56), and 4.37 (1.83). There was no advantage for the tailored condition compared with the placebo condition, including no differential rate of improvement (*b* = −0.03; 95% CI, −0.15 to 0.09; *t*_820_ = −0.49; *P* = .62; *d* = 0.12) or severity difference at 3-month follow-up (*b* = −0.07; 95% CI, −0.37 to 0.23; *t*_266_ = −0.48; *P* = .64; *d* = 0.11). Participants in the presession and postsession conditions showed faster improvement than those in the tailored condition (*b* = −0.22; 95% CI, −0.34 to −0.10; *t*_816_ = −3.62; *P* < .001; *d* = 0.94; and *b* = −0.17; 95% CI, −0.29 to −0.04; *t*_823_ = −2.65; *P* = .008; *d* = 0.72) and had lower severity at 3-month follow-up (*b* = −0.44; 95% CI, −0.73 to −0.14; *t*_257_ = −2.93; *P* = .004; *d* = 0.64; and *b* = −0.41; 95% CI, −0.72 to −0.11; *t*_257_ = −2.67; *P* = .008; *d* = 0.61). Finally, there were no statistically significant differences between presession and the postsession conditions for improvement rate (*b* = 0.05; 95% CI, −0.07 to 0.18; *t*_819_ = 0.79; *P* = .43; *d* = 0.21) or follow-up scores (*b* = 0.02; 95% CI, −0.29 to 0.33; *t*_270_ = 0.15; *P* = .88; *d* = 0.03).

**Table 3.** Raw Means and SDs of the Outcomes at Each Time Point

Scale and week	Score, mean (SD)			
	Presession (n = 38)	Postsession (n = 36)	Tailored (n = 40)	Placebo (n = 38)
Liebowitz Social Anxiety Scale total				
2	80.55 (14.86)	80.03 (19.72)	81.05 (17.67)	81.26 (18.53)
3	76.08 (15.93)	69.56 (18.85)	73.80 (16.57)	74.14 (21.24)
4	68.63 (16.72)	64.27 (22.75)	69.86 (20.10)	72.57 (23.32)
5	61.08 (18.95)	59.03 (22.97)	68.50 (20.96)	68.94 (23.01)
6	57.51 (22.24)	57.06 (24.76)	66.60 (22.39)	65.88 (25.16)
10	54.85 (21.90)	54.90 (25.86)	63.82 (24.42)	61.35 (25.37)
18	47.58 (22.37)	49.44 (25.58)	59.64 (24.32)	65.07 (27.96)
Social Phobic Disorders-Severity Form total				
2	5.47 (0.72)	5.31 (0.88)	5.50 (0.84)	5.47 (0.89)
3	5.26 (0.79)	5.03 (1.05)	5.08 (0.92)	5.11 (1.13)
4	4.95 (1.01)	4.42 (1.51)	4.68 (1.28)	4.89 (1.29)
5	4.26 (1.23)	4.06 (1.57)	4.69 (1.29)	4.75 (1.43)
6	4.11 (1.58)	3.97 (1.66)	4.62 (1.29)	4.45 (1.61)
10	3.84 (1.58)	3.82 (1.69)	4.39 (1.53)	4.16 (1.81)
18	3.21 (1.70)	3.50 (1.75)	4.12 (1.56)	4.37 (1.83)

Sensitivity analyses showed that none of the growth curve parameters differed significantly between the 2 outcomes. Further sensitivity analyses showed that site was a moderator of treatment (*F*_6,820_ = 4.05; *P* = .001 for site by condition by time). Rush University and The University of Texas at Austin sites had results identical to the overall reported results, whereas no DCS condition was significantly superior to placebo at Boston University.

In exploratory analyses, we tested whether the differences between the placebo condition and standard DCS administration conditions (presession and postsession) varied as a function of number of successful sessions: no effect was observed. We then examined whether differences between tailored and the presession and postsession conditions was accounted for by number of DCS doses in each treatment, by adding DCS doses as a factor associated with the growth curve parameters. Results showed that when treatments were equalized at 2.18 DCS doses (the mean number in the tailored condition), no differences existed between the tailored condition and the other 2 standard DCS conditions.

## Discussion

The present study sought to examine different DCS dosing regimens for augmenting exposure therapy of SAD. Contrary to the hypothesis, participants in the tailored DCS condition experienced less symptom improvement than participants assigned to either presession or postsession DCS administration. Moreover, the tailored DCS condition did not outperform the placebo condition. This failure for the tailored DCS administration strategy occurred against the backdrop of successful DCS augmentation with the most commonly applied presession administration of DCS, as well as the postsession DCS administration: both of these conditions offered more benefit than placebo and did not differ from each other. As such, this trial replicates a general benefit for DCS augmentation.^5^ Interestingly, the effect size estimates for presession and postsession DCS augmentation (*d* > 0.72) were above the effect sizes for such effects across the anxiety disorders (*g* = 0.25),^5^ but in line with estimates for the treatment of SAD specifically. In sum, although the current study extended previous work documenting the efficacy of DCS for facilitating exposure therapy efficacy,^5^ it failed to achieve the aim to develop a more effective application of this augmentation strategy.

The failure of successful DCS augmentation with a tailored approach, compared with the success of a standard approach, focuses attention on our criterion for exposure success. Keeping in mind clinical application and supported by pilot data,^9,10^ we developed a simple tailored approach that tied DCS administration to successful sessions defined as ending in a fear level 40 or lower. Although exposure success rates were consistent with expectations (and the assumptions of statistical power calculations^17^) and did not vary between conditions, it may be that the range of end fear scores associated with positive DCS effects was different for participants in the current study than those in our previous studies. Indeed, the cutoffs for end fear as a factor associated with the efficacy of cognitive enhancers has varied across studies. In support of this hypothesis, exploratory analysis from the current study confirms that session success as we defined it was not associated with the magnitude of the advantage of the standard DCS approach over placebo. It is also important to recognize that the effects for end fear in previous studies were associated not only with the positive effects of low end fear but also with the negative effects of high end fear, for which the range also remains unknown and might vary across and within individuals (longitudinally). Hence, aside from having missed the optimal low end fear range for augmentation, it is possible that the interfering effects of high end fear were absent in the current study, thus leaving minimal opportunity for the tailored strategy to offer both the facilitating (when the dose was given) and protective effects (when the dose was not given) we anticipated. Together, these observations call for the investigation of good learning during or success of an exposure session. Here, it is important to consider that a good learning index may involve measures other than (end) fear and that this index may vary across disorders as well as over the course of exposure therapy.

Exploratory analyses indicated that the greater effectiveness of DCS in the presession and postsession conditions compared with the tailored condition seemed to be accounted for by differences in the number of DCS doses (although it is important to note that verification of drug levels and target engagement was lacking). This result is consistent with the dose-response association observed in the meta-analysis.^7^

### Strengths and Limitations

This study has a number of strengths and limitations. To our knowledge, it is the first randomized clinical trial of an empirically informed tailored approach to DCS augmentation using high-quality methods (eg, double-blind design, state-of-the-art assessment and data analysis, and adequate statistical power). One limitation is that, because we opted to test the clinical strategy, we manipulated administration and not exposure success and, hence, cannot make any inferences regarding causal effects of exposure success on DCS efficacy. Another limitation is that our findings can only generalize to participants who completed at least 2 sessions of the 5-session protocol. Indeed, because we only administered study medication at the sessions that focused on exposure, we randomized participants at the beginning of session 2 and as a result were unable to include 17 participants who were eligible (but were lost before session 2) in the analyses.

## Conclusions

The current study showed that DCS, when administered before or after the session, augments exposure therapy for SAD, with evidence for clinically meaningful effects. Our findings did not support a tailored approach to DCS administration. In addition to underscoring the difficulty of accurately identifying sessions considered as successful and in need of memory augmentation, results hinted at the possibility that a sufficient number of DCS administrations is needed to produce its desirable augmentation effect.^7^ Accordingly, research is needed on (1) defining the characteristics of successful exposure sessions, (2) identifying individuals who benefit the most from augmentation of successful exposure sessions (eg, those who exhibit problems with fear extinction consolidation or suboptimal N-methyl-D-aspartate receptor function), and (3) determining optimal dosing of DCS.

## Notes

Supplement 1. **Trial Protocol**

Supplement 2. **Data Sharing Statement**
