# Supplementary material for: Dose Timing of D-Cycloserine to Augment Exposure Therapy for Social Anxiety Disorder: A Randomized Clinical Trial
Source: JAMA Netw Open. 2020 Jun 4;3(6):e206777. doi: 10.1001/jamanetworkopen.2020.6777 (PMC7273198; doi:10.1001/jamanetworkopen.2020.6777)
Supplement: Supplement 1. — Trial Protocol [file jamanetwopen-3-e206777-s001.pdf]

SECTION 2: SPECIFIC AIMS

Overview: This collaborative R34 application proposes a study that aims to provide the first step in the development of a simple algorithm for maximizing the efficacy of exposure therapy for social anxiety disorder with D-cycloserine (DCS). As part of this objective, we aim to evaluate the efficacy of tailored post-session DCS administration for enhancing the outcome of exposure therapy. This application was guided by the following observations:

- Despite being an empirically-supported first-line intervention for the anxiety disorders, exposure therapy is associated with substantial non-response rates, thus leaving ample room for improvement [7-10].
- Basic research on the therapeutic mechanism of exposure therapy - fear extinction - has indicated that fear extinction can be facilitated by the acute administration of the N-Methyl-D-Aspartate (NMDA) partial agonist, DCS [11].
- Clinical research evaluating the efficacy of acute pre-session administration of DCS for enhancing exposure therapy has yielded mixed results, with some studies showing strong effects [12-15], and others showing weak effects [16] or no effects at all [1, 5, 17].
- Basic research and clinical research aimed at explaining the variability in response to DCS augmentation have yielded findings that converge to suggest that DCS enhancement of exposure therapy depends on the success of exposure sessions [4, 18, 19]. Our research shows that positive effects of pre-session DCS administration are evident when subjects end training sessions with low fear but not evident when they end training sessions with elevated fear. These findings suggest that DCS should be administered only when sessions end with low fear, thus justifying consideration of post-session rather than pre-session DCS dosing.
- Basic research has demonstrated that post-session DCS administration is efficacious [20, 21]. Our initial clinical research findings suggest that post-session administration can be efficacious, but only when it follows successful exposure sessions (i.e., when patients end sessions with low fear) [4].

Aims: Building upon the aforementioned research, our objective is to optimize the application of DCS. To this end, we will test whether tailored post-session administration of DCS (i.e., only following successful sessions) will yield better outcomes than the strategy for DCS augmentation that has been utilized to date, namely pre-session DCS administration [22]. Because the difference between pre-session DCS augmentation and placebo augmentation has been variable across studies, we will also compare tailored post-session DCS-augmented exposure therapy against placebo-augmented exposure therapy. Finally, to determine if post-session administration must be tailored, we will also compare tailored post-session DCS administration vs. non-tailored post-session administration of DCS (i.e., following all sessions [irrespective of success]). We will enroll 156 patients with social anxiety disorder in a 5-session exposure therapy protocol and randomly assign them to one of the following arms (1) tailored post-session DCS administration; (2) pre-session DCS administration; (3) placebo; or (4) non-tailored post-session DCS administration. The primary outcomes will be short- and long-term improvements in social anxiety severity. The specific aims are as follows:

1. To examine the relative efficacy of tailored post-session DCS administration for augmenting exposure therapy. We expect that the tailored post-session DCS administration condition will outperform the pre-session DCS administration, placebo, and non-tailored post-session DCS administration conditions, respectively, at posttreatment, 1-month and 3-month follow-up.
2. To explore potential moderators (e.g., demographic characteristics, clinical characteristics, personality traits) of the efficacy of tailored post-session DCS administration for augmenting exposure therapy.

Unique to Each Site: Our team consists of experienced clinical trial investigators, including Drs. Mark Pollack (Rush University Medical Center; Rush), Stefan Hofmann and Michael Otto (Boston University; BU), Jasper Smits (University of Texas at Austin; UT). The proposed cross-site project management protocol will be identical to our previously completed collaborative linked R01 grant (MH075889; PI: Pollack, with contract to Smits and MH078308; PI: Hofmann). The results of our previous grant are currently published or in press [3-5]. Each of the sites will bring to this application complementary expertise. Rush (PI: Mark Pollack, M.D.) brings expertise in psychopharmacology and will oversee the quality assurance of the protocol for administering and measuring safety and tolerability of DCS. BU (PI: Stefan Hofmann, Ph.D.) brings expertise in CBT for SAD and will oversee the training and supervision of study therapists. UT (PI: Jasper Smits, Ph.D.) brings expertise in CBT, assessment of SAD, and statistical analyses (David Rosenfield, Ph.D.; consultant), and will provide training and supervision of study therapists. This application is the logical next step in the study of DCS. It provides an important innovative move toward the realization of personalized medicine by providing the first step in the eventual development of an algorithm for administering DCS in CBT with the goal of maximizing the efficacy and cost-effectiveness of therapy for anxiety disorders, which are the most prevalent mental conditions, making this a project of potentially high public health significance.

SECTION 3: RESEARCH STRATEGY

3A: SIGNIFICANCE

3A.1: Anxiety Disorders: A Major Public Health Problem.

Epidemiological studies indicate that anxiety disorders are the most prevalent class of mental disorders with 12-month and lifetime prevalence rates of 18.1% and 28.8%, respectively [23, 24]. Anxiety disorders have been associated with increased health care utilization and lost employment [25], and have an annual cost to the United States economy of at least $42 billion dollars [26].

3A.2: Exposure-Based Treatment: Efficacious But Leaving Ample Room for Improvement.

The last three decades have seen a tremendous amount of research focusing on the development of exposure-based treatments for the anxiety disorders. Exposure therapy has demonstrated clear efficacy for the anxiety disorders [8-10], offering clinically meaningful advantages over psychological placebo conditions [7], and showing improvements in symptoms comparable to established pharmacotherapies [27]. However, many patients who receive exposure therapy either fail to respond or continue to experience residual symptoms following treatment discontinuation [8-10]. For example, large clinical trials of exposure-based treatment efficacy for the various anxiety disorders have yielded non-response rates of up to 49% for social anxiety disorder [8], 38% for obsessive-compulsive disorder [10], and 36% for panic disorder [9]. Accordingly, the agenda for exposure therapy research has shifted to the development of strategies to enhance the effectiveness of exposure therapy.

3A.3: DCS and the Augmentation of Exposure-Based Treatment: Strong Theoretical Model.

In a particular success of translational research[28], the application of D-cycloserine (DCS) as an augmentation strategy was the product of animal research on the brain circuits underlying fear extinction [11] – the theoretical model for exposure-based therapy. Extinction learning appears to be modulated by activity of the glutamatergic N-Methyl-D-Aspartate (NMDA) receptor in the amygdala. Inhibitors of this activity appear to block the retention of extinction learning [29], and likewise, partial agonists, like DCS, enhance this learning [30]. Specifically, it appears that DCS enhances the consolidation of the new learning that occurs during extinction; augmentation effects continue to be present when DCS is administered within several hours after extinction [20].

These exciting preclinical findings were first extended to the clinic in a placebo-controlled trial by Ressler et al. [14]. Height phobic patients were given a single dose of study medication prior to each of two exposure sessions. Exposure was conducted using a virtual reality apparatus, and the use of only two exposure sessions mirrored animal study designs. Ressler et al. [14] found that DCS given prior to exposure significantly enhanced extinction learning, and this effect was evident at both 1 week and 3 months follow-up.

3A.4: DCS and the Augmentation of Exposure-Based Treatment: Mixed Findings.

The successful trial by Ressler and associates (2004) was followed by a number of other positive studies of pre-session DCS augmentation starting with a small-scale trial by our group focusing on social anxiety disorder [13]. In that study, we randomized 27 patients with SAD to 50 mg DCS or pill placebo. DCS was administered one hour before the final four sessions of a 5-session protocol of weekly treatment. During these four sessions, patients completed social exposure exercises. Patients who had received DCS achieved significantly more benefit as evaluated at acute and one-month follow-up assessments. This small study was subsequently replicated by Guastella et al. [15] with a larger sample size (N = 56); similar benefit for DCS administration was found. We have also reported similar positive findings for the application of DCS to the treatment of panic disorder [12]. However, at the same time, there have been trials showing weak [3, 16] or no effects [1, 5, 17, 31] of DCS enhancement of exposure therapy for the anxiety disorders. For example, we recently completed the largest trial of DCS augmentation of exposure therapy completed to date (N = 169), and found that, on average, patients receiving a full 12-session course of CBT that included 5 DCS-augmented sessions did not evidence significantly higher response and remission rates relative to patients receiving placebo-augmented CBT, despite showing a greater rate of improvement [3].

3A.5: Explaining Variability in DCS Efficacy: A Case for Tailored Post-Session Administration of DCS.

Theoretically, positive effects of DCS on exposure therapy outcome are expected to be dependent on the degree to which adequate extinction learning has occurred [11]. Indeed, DCS has been conceptualized as a memory enhancer, aiding the consolidation (i.e., retention) of what was learned during extinction training [11, 32]. Consistent with this account, a number of animal studies investigating the limits of DCS augmentation effects indicate that augmentation effects are achieved only with animals that demonstrate extinction at the time the DCS is administered. For example, Weber and colleagues [18] separated animals into those that had and those that had not demonstrated extinction learning; only the former group showed DCS augmentation effects. Likewise, in a re-analysis of a null-finding study [33] of DCS augmentation, Bouton and colleagues [19] performed a

median split of their sample based on the extinction effects during the drug session; again, a significant DCS augmentation effect was seen only for the animals that had demonstrated stronger extinction learning.

Recently, we demonstrated that these moderator effects observed in animal studies of DCS enhancement of fear extinction translate to DCS enhancement of exposure therapy (see pilot studies below). In the first study [2], we reanalyzed the data from our recently completed trial involving patients with social anxiety disorder (N = 169) and found, consistent with expectations, that relative to patients receiving placebo, patients receiving DCS evidenced significantly greater clinical improvement when they reported low fear at the end of their previous exposure session. In contrast, when exposure end fear was high, patients receiving DCS exhibited less clinical improvement at the following session than patients receiving placebo. Similarly, patients receiving DCS only evidenced lower clinical severity at posttreatment than patients receiving placebo when the average end fear at each session was in the mild to moderate range (i.e., posttreatment CGI-S difference between DCS and Placebo for patients with an average end fear of 30 = -1.35). Finally, these moderating effects of exposure success as indexed by end fear were not better accounted for by within-session extinction, suggesting that end fear (as opposed to a change in fear) is a good index for predicting DCS augmentation effects. In the second study [4], we tested whether these moderating effects of session end fear on DCS efficacy would be evident when DCS is administered post-session (instead of pre-session). The main outcome analysis for the parent trial for this re-analysis had revealed no evidence of post-session DCS augmentation of virtual reality therapy in height phobics (N=29) on average [5]. However, consistent with predictions, the effects of post-session DCS administration on clinical improvement was moderated by the level of fear experienced just prior to concluding exposure sessions. Patients receiving DCS exhibited significantly greater improvement in symptoms relative to patients who received placebo when subjective fear was low at the end of the exposure (posttreatment CGI-I difference between DCS and Placebo for patients with an average end fear of 0 = 1.0). In contrast, when end fear was still elevated, patients receiving DCS improved less compared to those receiving placebo (posttreatment CGI-I difference between DCS and Placebo for patients with an average end fear of 36 = -.84).

These findings are consistent with studies showing that DCS not only augments extinction learning but it also enhances reconsolidation of fear memory in animals [34] and humans [1]. In contrast, NMDA antagonists impair the reconsolidation of fear memories [35-37]. Therefore, if fear does not decrease during exposure, fear memory reconsolidation may occur and DCS may facilitate this counter-therapeutic process. In other words, DCS can make “good” exposures better and “bad” exposures worse. Together, these observations suggest that the effective application of DCS may warrant judicious use, i.e., limiting the administration to exposure sessions that are characterized by low end fear (adequate extinction learning; exposure success). This is arguably best achieved by administering DCS post- instead of pre-session. Support for tailored post-session administration of DCS (i.e., only following successful session) over pre-session dosing (which has been the strategy in all but one clinical trial [4, 5] completed to date), comes from (1) animal studies that have documented success with post-session DCS administration up to 2 hours following training [20, 21] and (2) pilot work from our group showing that post-session administration of DCS is efficacious when linked to successful exposure sessions (adequate extinction learning) [4].

3B: INNOVATION

3B.1: Initial Step Toward the Development of an Algorithm for Prescribing DCS.

The utilization of DCS in conjunction with exposure-based CBT represents a revolutionary approach to combination treatment that follows a translational research agenda. Rather than combining two different modalities of treatment, each aimed at anxiolysis, DCS augmentation is aimed at the facilitation of the learning processes underlying exposure-based therapy [22]. It should be noted that DCS is not an anxiolytic, but instead acts as a cognitive enhancer, augmenting the learning processes that occur during exposure therapy. This unique and novel approach presents a genuine paradigm shift in treatment research, translating preclinical neuroscience directly into novel clinical applications. For the first time, a pharmacological agent that does not have any independent anxiolytic properties is being used to enhance one of our most effective psychological treatments for anxiety disorders based on a known mechanism.

Initial clinical studies investigating DCS augmentation have employed pre-session dosing, administering DCS one hour prior to exposure sessions. Findings have accumulated to suggest that this blanket approach to the administration of DCS may not yield the best outcomes for patients. Indeed, acting as a cognitive enhancer, DCS’ positive effects appear dependent upon success of exposure therapy sessions, defined as low subjective fear prior to concluding the exposure exercise. In this protocol, we have a design (as well as the expertise and infrastructure) that allows for the evaluation of tailored post-session dosing, and also explore potential moderators of this effect. Accordingly, it is expected that the findings of this research will aid the development of an algorithm for the effective application of DCS in exposure treatment for anxiety disorders.

3B.2: What is the Relevance of this Application to the NIMH RDoC Initiative?

The over-emphasis of symptoms and syndromes for the nosology and treatment of anxiety disorders has presented an obstacle for further research and therapy. As noted by Insel et al. [38], medical history has taught us that disorders that used to be considered unitary based on clinical presentation have been shown to be heterogeneous based on laboratory tests or treatment studies, whereas syndromes that may appear clinically distinct may result from the same etiology. Moreover, the same abnormal mechanisms may be involved in many different anxiety disorders, while on the other hand, not all patients with the same anxiety disorder necessarily show the same abnormality. This suggests that there may be common mechanisms involved across different disorders. However, these fundamental mechanisms are not yet well understood.

Although we rely on the current diagnostic system, our study is consistent with the Research Domain Criteria (RDoC) framework, which encourages the exploration of patient characteristics that might enable us to group patients into more meaningful categories than existing diagnostic categories. The RDoC construct of interest of the proposed study is Acute Threat ("fear"), in the domain of Negative Valence Systems. Although the objective is not designed to test RDoC per se, the results of the proposed study are fully consistent within the RDoC framework because the results will provide valuable data toward personalizing treatment. More specifically, our study will translate knowledge from basic animal research about extinction learning to exposure therapy in humans in order to identify clinical and person-specific predictors of treatment response. Thus, the results of this study not only provide concrete treatment guidelines for treating anxiety disorder, but they also add valuable information for future nosology systems and associated interventions.

3B.3: Need for the Proposed Pilot Trial (R34).

This application was submitted in response to FOA "Collaborative R34s for Pilot Studies of Innovative Treatments in Mental Disorders (Collaborative R34)", which aims to support the early phases of intervention development or adaptation, protocol development and preliminary pilot testing to support a full-scale (e.g., R01) efficacy or effectiveness study, and innovative services research. The present application seeks funding for the first in a series of studies aimed at establishing an algorithm for the prescription of DCS in the treatment of anxiety disorders and related psychopathology. Specifically, building upon the findings of early studies evaluating the efficacy of DCS augmentation, this study will test, in an adequately powered RCT involving patients with social anxiety disorder, whether tailored post-session administration of DCS outperforms pre-session administration (the prescription strategy applied and tested in most studies completed to date). As such, the present application is the first investigation of an empirically-informed adaptation (i.e., tailored post-session dosing) of an existing intervention protocol (i.e., pre-session dosing). Support for the hypotheses to be tested in the present application and results from our proposed exploratory moderator analyses will provide the basis for a follow-up R01 study aiming to develop an algorithm for the application of DCS for augmenting cognitive behavioral treatment to ameliorate anxiety disorders more broadly (i.e., beyond social anxiety disorders) or the clinical dimensions of psychopathology embedded in these diagnostic entities.

Our rationale for proposing a 3-site collaborative approach is two-fold: (1) Increase sample size and accelerate recruitment. Achieving the aims of the proposed study requires a sample size of 156 (see power section below), which, based on our previous work, justifies utilizing 3 sites (see recruitment section below); (2) Increase sample diversity. As we demonstrated in our previous linked application, utilizing three sites in different areas of the US resulted in the expected increase in diversity in our sample, and allowed for testing of the generalizability of study findings.

3C: APPROACH

3C.1: Preliminary Studies.

Our group was the first to demonstrate the DCS augmentation effect of exposure therapy for social anxiety disorder [13] and we have since conducted a number of studies of DCS augmentation across anxiety disorders [1, 3, 5, 12], with recent attention to moderators of efficacy [2, 4]. We will review here only the studies that are most relevant to the current application.

Augmentation of exposure therapy for social anxiety disorder with D-cycloserine. Arch Gen Psychiatry. 2006;63:298-304. Participants were 27 patients with a principal DSM-IV diagnosis of social anxiety disorder (SAD); 51.9% had a generalized subtype SAD and 40.7% had additional mood or anxiety disorder comorbidity.

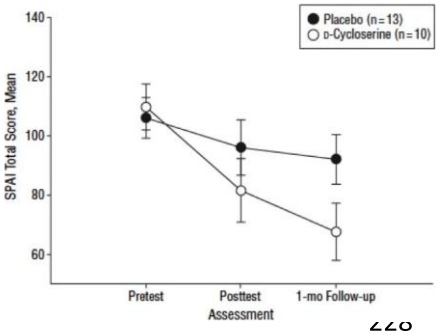

Participants were randomly assigned to receive either exposure group therapy plus DCS (50 mg) or exposure group therapy plus pill placebo prior to the last 4 sessions of a 5-session protocol of exposure-based CBT; the first session was psychoeducational. The exposure sessions consisted of repeated exposure to challenging public speaking situations in front of the other group members, plus cognitive restructuring. There were advantages for DCS vs. placebo augmentation in the medium to very large range from pre to post-treatment and from pre-treatment to follow-up (see Figure on the left).

Efficacy of D-cycloserine for enhancing response to cognitive-behavior therapy for panic disorder. Biol Psychiatry. 2010;67:365-70.

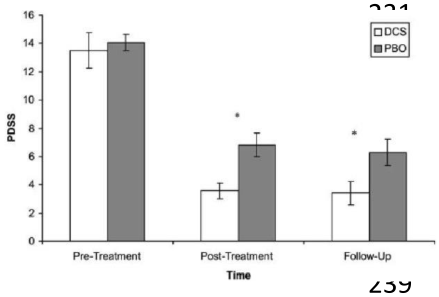

In this study, we randomized 31 outpatients with DSM-IV panic disorder with or without agoraphobia to pre-session DCS or placebo administration during sessions 3 to 5 of a 5-session CBT protocol for panic disorder. Participants who received DCS versus placebo had better outcomes on the PDSS and global severity of disorder and were significantly more likely to have achieved clinically significant change status (77% vs. 33%; see Figure on the left).

D-cycloserine as an augmentation strategy with cognitive-behavioral therapy for social anxiety disorder. Am J Psychiatry. 2013;170:751-58. This study was a linked 3-site randomized controlled trial

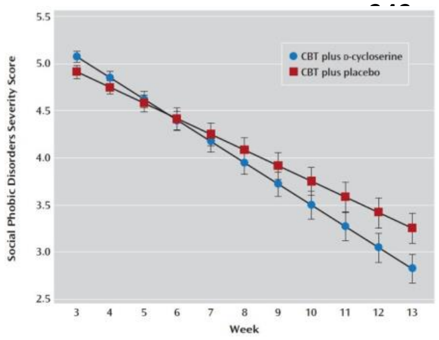

(R01MH07830; R01MH075889) evaluating the efficacy of pre-session DCS administration for augmenting a standard 12-session CBT protocol for SAD. Participants in this trial were 169 patients with a principal DSM-IV diagnosis of generalized SAD who were free of psychotropic medications. All participants received 12 sessions of CBT and were randomly assigned to either 50 mg of DCS or placebo 1-hour prior to sessions 3-7. D-cycloserine-augmented and placebo-augmented CBT were associated with similar completion rates (87% and 82%), response rates (79.3% and 73.3%), and remission rates (34.5% and 24.4%) at the posttreatment assessment; response and remission rates were largely maintained at the follow-up assessments. Relative to patients receiving placebo, those receiving D-cycloserine evidenced a 24%–33% faster rate of improvement in symptom severity and remission rates relative to placebo during the treatment phase (see Figure on the left). Further, at posttreatment, patients who received d-cycloserine showed lower global illness severity scores than patients who received placebo (see Figure on the left). There were no significant between-group differences on the LSAS or response and remission rates at posttreatment.

A randomized placebo-controlled trial of D-cycloserine and exposure therapy for posttraumatic stress disorder. J Psychiatr Res. 2012;46:1184-90. As part of this study, we recruited 26 veterans of the Iraq and Afghanistan wars who had a primary diagnosis of PTSD. Patients were randomly assigned to exposure therapy plus DCS (n = 13) or exposure therapy plus placebo (n = 13). Subjects received 50 mg of DCS or placebo 30 minutes prior to sessions 2-5. The results showed that DCS was associated with poorer outcome than placebo from pre- to post-treatment. Of note, the two arms had equivalent fear ratings prior to the initial exposure session (when DCS was first administered), but patients in the DCS arm reported significantly higher fear at the end of each augmented session relative to patients in the placebo condition. These findings are consistent with the possibility the lack of DCS enhancement observed in this trial was due to insufficient extinction learning.

D-cycloserine enhancement of exposure therapy for social anxiety disorder depends on the success of exposure sessions. J Psychiatr Res. In Press. Using the data from our large-scale social anxiety disorder trial, we tested the hypothesis that DCS enhancement of fear extinction would be specific to successful exposure sessions. Participants provided fear ratings at the beginning and just before the end of exposure exercises. Independent raters, blind to group assignment, administered the clinical global impression improvement and severity scales at each session and at posttreatment. End Fear ratings ranged from 0 to 100 (M=49.5; SD=19.2), and Beginning Fear ranged from 10 to 100 (M=66.7; SD=17.8). Average End Fear across the 5 augmented sessions ranged from 5 to 83.8 (M=49.2; SD=15.5) and Average Beginning Fear across the 5 augmented sessions ranged from 30 to 100 (M=66.7; SD = 14.5). Mixed-effects analyses revealed that, relative to patients receiving placebo, patients receiving DCS evidenced significantly greater clinical improvement when they reported low fear at the end of their previous exposure session (see Figure 1 on the left). In contrast, when exposure end fear was high, patients receiving DCS exhibited less clinical improvement at the following session than patients receiving placebo. Similarly, patients receiving DCS only evidenced lower clinical severity at posttreatment than patients receiving placebo when the average end fear was in the mild to moderate range (see Figure 2 on the left). Finally, these moderating effects of exposure success as indexed by end fear were not better accounted for by within-session extinction.

Figure 1. *Per-session Effects*: DCS interacts with End Fear to Predict *Improvement* at the Next Session

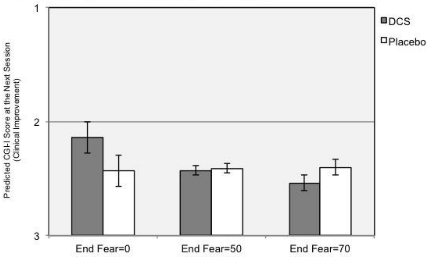

Figure 2. *Cumulative Effects*: DCS interacts with Average End Fear to Predict *Severity* at Posttreatment

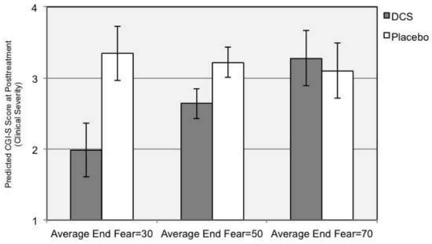

D-cycloserine enhancement of fear extinction is specific to successful exposure sessions: evidence from the treatment of height phobia. Biol Psychiatry. 2013;73:1054-8. We conducted a re-analysis of existing data to examine whether the effects of post-session DCS administration on clinical outcome would vary as a function of exposure success. In a clinical trial, patients with height phobia received two sessions involving 30 minutes of virtual reality exposure therapy and were randomly assigned to a pill placebo (N=14) or 50 mg of DCS (N=15) immediately after each session. Mixed-effects regression analysis showed that the effects of DCS administration on clinical improvement was moderated by the level of fear experienced just prior to concluding exposure sessions (see Figure on the left). Patients receiving DCS exhibited significantly greater improvement in symptoms relative to patients who received placebo when subjective fear was low at the end of the exposure. In contrast, when end fear was still elevated, patients receiving DCS improved less compared to those receiving placebo.

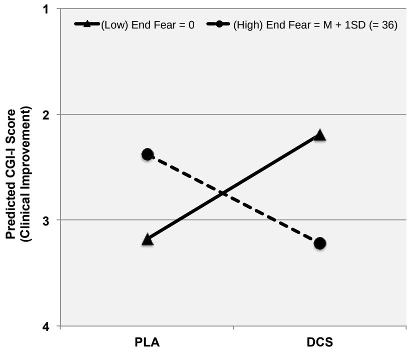

Summary of Pilot Studies. In sum, it has been shown that DCS can augment exposure therapy for a range of different anxiety disorders. However, post-hoc analyses suggest that DCS may only enhance exposure treatment when applied after successful sessions, and may have detrimental effects when administered after inadequate/unsuccessful exposures. This is consistent with preclinical data showing that DCS not only augments extinction learning, but also enhances fear-reconsolidation. Therefore, DCS is of limited clinical value - and may even be harmful - if it is routinely administered before exposure treatments. For DCS to be a clinically valuable augmentation strategy, it will be essential to examine the effect of post-session administration with the goal of specifically augmenting only successful (i.e., tailored) exposure sessions. Thus, the primary objective of this application is to determine the efficacy of tailored post-session DCS administration for enhancing exposure therapy by using an established proof-of-concept design. This study objective calls for a direct comparison between exposure treatments with conventional pre-session DCS administration, tailored post-session DCS administration, non-tailored post-session DCS administration, and placebo administration.

3C.2: Experimental Design.

We propose to enroll 156 patients (52 per site) into a 5-session CBT protocol and randomly assign them to 1) Tailored post-session DCS administration: pre-session PBO and selective post-session DCS administration during sessions 2-5; (2) Pre-session DCS administration: pre-session administration of 50 mg of DCS and post-session administration of pill placebo (PBO) (during sessions 2-5); (3) Placebo administration; pre- and post-session administration of PBO (during sessions 2-5) or (4) Non-tailored post-session DCS administration: pre-session PBO and post-session DCS administration (during sessions 2-5; see Table 1). In order to meet the enrollment target and ensure a diverse and representative sample, this study will be completed at three study sites (Rush, BU, and UT), all following an identical study protocol. Our team (Drs. Pollack, Hofmann, Smits, Otto, and Rosenfield) has a long history of successful, NIMH-funded collaborations in the area of anxiety disorders with a particular focus on translational research and the treatments for social anxiety disorder. The three sites have complementary areas of expertise, which minimizes experimenter effects and other biases.

Table 1. Conditions

Conditions	Pre-session Pill	Post-session Pill
1. Tailored Post-session DCS	PBO	DCS After Successful Sessions<br>PBO After Non-successful Sessions
2. Pre-session DCS	DCS	PBO
3. Placebo	PBO	PBO
4. Non-tailored Post-session DCS	PBO	DCS

3C.2a: Design Considerations.

Why utilize a 4-cell design? The primary aim of the current application is to test whether tailored post-session DCS administration outperforms pre-session DCS administration. This aim justifies inclusion of conditions 1 and 2 (see Table 1). Because the efficacy of pre-session DCS administration (relative to placebo) has been variable across studies to date, it is important to include a tailored post-session DCS administration vs. placebo comparison, thus justifying condition 3 (see Table 1). Finally, in order to determine whether post-session administration has to occur in a tailored fashion rather than unselectively (i.e., irrespective of end fear levels), we included a non-tailored post-session DCS administration condition (condition 4; see Table 1).

Why administer 50 mg of DCS? DCS has a generally benign side effect profile when used at typical doses for CBT augmentation (i.e., 50 mg). We identified three early studies (total N=62) of DCS administration to healthy controls [39, 40]. All studies employed placebo-controlled, single-pill administrations of DCS, with the dosages ranging from 15 mg to 500 mg per administration and 3 to 7 days in between administrations. No adverse effects were reported. This benign perspective on the side effects associated with DCS should be contrasted with its side effect profile when administered in much larger doses. DCS has been FDA-approved for approximately 20 years for the treatment of tuberculosis, and is generally chronically dosed at 250-500 mg delivered twice daily. It is primarily renally excreted with a half-life of 10 hours. When using high doses, side effects associated with these chronic dosing schedules include drowsiness, headache, confusion, tremor, vertigo, memory difficulties, paresthesias, and seizure. Smaller doses produce very little, if any, side effects. In fact, studies that have used 50 mg doses report that participants are unable to differentiate between placebo and DCS. Accordingly, the use of 50 mg dose of DCS, offered only acutely, may be a particularly effective strategy for minimizing side effects. Also, Ressler et al. [14] found no difference between the effects of 50 mg or 500 mg in enhancing exposure-based treatment for the fear of heights. Further, our group has shown that 50 mg of DCS facilitates the efficacy of exposure-based therapy in SAD [2-4] and panic disorder [12], with an independent replication of this effect in a larger sample of SAD patients [15]. Therefore, we will utilize procedures consistent with previous successful trials, and administer 50 mg of DCS.

Why utilize our 5-session CBT for SAD? This protocol has been used in previous proof-of-concept studies examining the efficacy of DCS as an augmentation strategy for exposure procedures in a clinical population [13, 15], making it particularly suited as a standard for (1) the investigation of post-session DCS effects, and (2) consideration for ultimate dissemination for clinical treatment.

Why administer DCS on 4 occasions during a 5-week intervention? Given concerns about tolerance from too frequent or too many doses [22], we tailored the DCS application for the present study to the administration frequency that has been found to be successful in past research. We decided to target sessions 2-5 for DCS augmentation because these sessions include the crucial training (i.e., exposure) for extinction.

Why use end fear of 40 as the threshold for tailored post-session DCS administration? This cut-off was established using the data collected as part of our large-scale trial (N=169) of DCS enhancement of exposure in social anxiety disorder [2]. The threshold at which DCS had an advantage over placebo in that trial

was an end fear subjective units of distress (SUDS) rating of 47. Indeed, at 47, DCS and placebo did not differ and at 70, placebo significantly outperformed DCS. At end fear SUDS ratings below 47, there was an advantage of DCS over placebo that increased linearly as the end fear SUDS ratings decreased. Our recently completed study [6] using the same intervention that we will use in the present investigation, showed that, during the four augmented sessions, 85% of patients had at least 1 end fear SUDS rating of 40 or below, and 67.5% had at least 2 end fear SUDS rating of 40 or below.

Is the variability in end fear ratings sufficient to justify the inclusion and examination of both tailored and non-tailored post-session DCS administration? End fear ratings during the 5 augmented exposure sessions in our recently completed large trial of DCS enhancement for exposure therapy in social anxiety disorders [3] ranged from 0 to 100 (scale: 0-100), with a mean of 49.5 (SD=19.2). Similarly, end fear ratings during a the 4 augmented exposure sessions (in a protocol identical to that of the current application) in our recently completed preliminary trial (N=40) evaluating yohimbine enhancement of exposure therapy [6] ranged from 0 to 90 (scale: 0-100), with a mean of 39.1 (SD=17.7).

3C.3: Sample

Consistent with our previous trials, we propose the following study criteria to balance scientific rigor, feasibility, and ethical/human subjects concerns. We recruited experienced clinical trial investigators (Drs. Gail Steketee, Murray Stein, and Sabine Wilhelm) to serve on our DSMB. Drs. Steketee and Stein have also served on the DSMB of our previous trial. Inclusion criteria: (1) Male or female outpatients > 18 years of age with a primary psychiatric diagnosis (designated by the patient as the most important source of current distress) of generalized social anxiety disorder as defined by DSM-5 criteria. (2) A total score > 60 on the LSAS. (3) Physical examination and laboratory findings without clinically significant abnormalities. (4) Willingness and ability to participate in the informed consent process and comply with the requirements of the study protocol. Exclusion criteria: (1) A lifetime history of bipolar disorder, schizophrenia, psychosis, delusional disorders or obsessive-compulsive disorder; an eating disorder in the past 6 months; organic brain syndrome, mental retardation or other cognitive dysfunction that could interfere with capacity to engage in therapy; a history of substance or alcohol abuse or dependence (other than nicotine) in the last 6 months or otherwise unable to commit to refraining from alcohol use during the acute period of study participation. (2) PTSD within the past 6 months. Entry of patients with other mood or anxiety disorders will be permitted if the SAD is judged to be the predominant disorder, in order to increase accrual of a clinically relevant sample. Patients with significant suicidal ideation (MADRS item 10 score > 3) or who have enacted suicidal behaviors within 6 months prior to intake will be excluded from study participation and referred for appropriate clinical intervention. (3) Patients must be off concurrent psychotropic medication (e.g., antidepressants, anxiolytics, beta blockers) for at least 2 weeks prior to initiation of randomized treatment. (4) Significant personality dysfunction likely to interfere with study participation. (5) Serious medical illness or instability for which hospitalization may be likely within the next year. (6) Patients with a current or past history of seizures. (7) Pregnant women, lactating women, and women of childbearing potential who are not using medically accepted forms of contraception (e.g., IUD, oral contraceptives, barrier devices, condoms and foam, or implanted progesterone rods stabilized for at least 3 months). (8) Any concurrent psychotherapy initiated within 3 months of baseline, or ongoing psychotherapy of any duration directed specifically toward treatment of the SAD is excluded. Prohibited psychotherapy includes CBT or psychodynamic therapy focusing on exploring specific, dynamic causes of the phobic symptomatology and providing management skills. General supportive therapy initiated > 3 months prior is acceptable. (9) Prior non-response to adequately-delivered exposure (i.e., as defined by the patient's report of receiving specific and regular exposure assignments as part of a previous treatment). (10) Patients with a history of head trauma causing loss of consciousness, seizure or ongoing cognitive impairment.

3C.4: Procedures

3.C.4a. Timeline. During the study start-up phase (months 1-4), we will devote effort to training of all personnel and preparation of study books and materials. Enrollment of participants will occur during months 4 through 31. This schedule will allow for sufficient time to complete all follow-up assessments by month 34. Data analysis will be completed during months 32 to 36. We have achieved this accrual rate (~2 patients per month/per site or 6 per month total) in ongoing and past three-site trials.

3C.4b: Recruitment. Recruitment strategies will be based upon those successfully employed in our previous and ongoing studies. Specifically, in addition to relying on referrals from our respective clinics, we will utilize newspaper and radio advertisements posted in the general and university community. Given the enrollment rate in previous studies [3], we anticipate that the enrollment of 156 patients (52 per site) over three years is well within the capabilities of the sites.

3C.4.c: Determining Eligibility.

Telephone prescreen. A telephone prescreen will be conducted for all potential participants. Persons who appear eligible will be asked to come to the study site for diagnostic screening.

Screen Visit 1: Diagnostic screening. Upon arrival, participants will receive an informed consent form explaining the details of the study, potential benefits and risks of participation, and the procedures they will undergo if they choose to participate. If the individual chooses to sign the informed consent, he or she will begin the psychiatric evaluation process, which will include the Structured Clinical Interview for DSM-5 (SCID) and the Liebowitz Social Anxiety Scale (LSAS) to evaluate the presence of psychiatric inclusion and exclusion criteria. The interview will also allow for assessment of primary and secondary diagnoses if applicable (see 6C1.a for integrity of diagnostic assessment).

All clinician-rated outcomes will be provided by Independent Evaluators (IEs) who are blind to treatment assignment. The IEs for the proposed study will be Ph.D. or Master's-level diagnosticians who have previous research experience with structured interviewing. A two-level system will be used to maintain the reliability of diagnoses and of other clinical ratings in the study. At least 10% of all intakes will be interviewed by two independent clinicians. Furthermore, all clinical assessments will be digitally recorded. Each month, an independent evaluator (IE) will listen to an assessment performed by another IE associated with the study and independently complete the assessment instruments. These duplicate ratings will be used both to calculate kappa coefficients and for supervision. Differences between raters will be discussed during supervision to identify reasons for disagreement and improve inter-rater reliability. These procedures will help to ensure that IEs refine their diagnostic skills and will also establish common guidelines for ongoing use in diagnostic decision-making. Inter-rater agreement will be assessed via evaluation of digital recordings of diagnostic interviews (as described above). Inter-rater reliability (kappa) will be calculated for all anxiety disorders and MDD.

Screen Visit 2: Medical screening. A study physician will review the patient's medical history and conduct a complete physical examination, ensuring that there are no medical conditions that preclude study participation. Safety evaluations will also include laboratory tests (CBC, chemistry profile, thyroid function test, and urinalysis). A urine pregnancy test will be performed on all female participants of childbearing potential at pre-treatment. The physician will also discuss the potential side effects of DCS with potential participants.

3C.4d: Randomization. The project biostatistician, Dr. David Rosenfield, will oversee the randomization. We will block-randomize patients by site, using variable-sized permuted block-randomization (block sizes will vary from 4 to 12). Randomization for each site will be calculated before the first subject is run, and the condition assignment be put in numbered envelopes. Envelopes will be opened and randomization will occur at the inception of the second session. Prior to data analyses, Dr. Rosenfield will check the balance of randomization and control for any factors that are imbalanced.

3C.4e: CBT Protocol. Consistent with our previous study [13] and an independent replication trial [15], we will use a 5-session version of a group CBT protocol with 4-6 patients and 2 therapists per group emphasizing repeated exposure practices. Session 1 involves an introduction and orientation to the CBT model. Sessions 2-5 emphasize repeated exposure tasks, which consist of role-play activities to confront fearful situations in a group setting while disputing cognitive distortions (coupled with the fading of safety behaviors). Therapists are PhDs or advanced, trained doctoral students supervised by Drs. Hofmann and Smits. In the first session (60 minutes), patients are provided with a model of social anxiety disorder and its treatment with exposure therapy. In sessions 2-5 (90 minutes each), patients will be introduced to the social exposure procedures. The exposure practices of increasing difficulty consist of giving speeches about topics chosen by the therapists in front of the other group members, confederates, and a video camera. Patients' videotaped performance will then be reviewed. At the conclusion of each exposure session, patients will be encouraged to continue to apply home-practice strategies (such as giving speeches in front of a mirror). Continued practice of the interventions will be considered part of treatment, and patients will be asked to refrain from alternative treatment for four weeks following completion of the last treatment session.

3C.4f: Pill (D-cycloserine or Placebo) Administration Protocol. Study capsules will be compounded by Abrams Royal Pharmacy in Dallas, TX containing: (a) 50 mg DCS (derived from Seromycin 250 mg capsules) and polyethylene glycol 3350 powder or (b) polyethylene glycol 3350 powder (Placebo). In order to maintain the blind, we will implement the following procedures: (1) All capsules will be identical in appearance; (2) All capsules will be administered by research staff blind to study condition and not involved in the treatment or assessment of study participants; (3) The pharmacist will fill three bottles (one pill each) for each patient for each session according to the schedule in Table 2. Research staff will administer a pill from Bottle 1 to each participant one-hour before the session and administer a pill from either Bottle 2 or 3 immediately after the

Table 2. Pill Administration Procedures			
	**Pre-Session**	**Post-Session**	
		After ***Successful*** Sessions	After ***Unsuccessful*** Sessions
**Conditions**	**Bottle 1**	**Bottle 2**	**Bottle 3**
1. Tailored Post-session DCS	PBO	DCS	PBO
2. Pre-session DCS	DCS	PBO	PBO
3. Placebo	PBO	PBO	PBO
4. Non-tailored Post-session DCS	PBO	DCS	DCS

session. The selection of Bottle 2 vs. Bottle 3 will be guided by the end fear level. Specifically, a pill from Bottle 2 will be administered when end fear is ≤40, whereas a pill from Bottle 3 will be selected when end fear is >40; (4) Both therapist and staff member will be blind to the contents of the bottles and additionally, the therapist will be blind to which post-session bottle is employed.

3C.4g: Assessment Visits. The schedule of assessment visits (see Table 3) is such that it allows us to (1) compare outcomes in the present study to that observed in previous studies, and (2) carefully explore potential moderators.

3C.5: Assessments.

Screening. Eligibility Screen. This telephone-screening questionnaire will assess inclusion and exclusion criteria. Demographics. Participants will be asked to provide standard demographic information (age, sex, race/ethnicity, level of education, cohabitation status) as well as history of medical problems. Psychiatric History. Diagnostic exclusions and lifetime prevalence of Axis I diagnoses will be determined by the Structured Clinical Interview for DSM-5 (SCID) during the screening visit. The diagnostic interview will be administered by study investigators or trained graduate student-level clinicians and will be supervised by the PIs.

Laboratory Testing. Urine toxicology screens, AlcoBreath tube, and pregnancy testing will be performed at each site with the results considered in evaluation of the patient's clinical history of substance use or abuse. The urine toxicology screen includes assessment of the following agents: amphetamines, benzodiazepines, barbiturates, cocaine metabolites, marijuana, narcotics, and sedative hypnotics. Patients evidencing impairment or other clinical signs or symptoms of substance abuse (e.g., odor of alcohol on breath, slurring of speech) will be clinically evaluated including toxicology screens at the time if warranted. Their appropriateness for continued treatment in the study protocol or need for alternative services will be considered and if medically indicated, patient will be discontinued from the study and will receive referral for necessary clinical treatment. Laboratory assessment will be performed including CBC, chemistry profile, thyroid screen, and urinalysis, as part of the general physical medical evaluation of patients at screen, including medical history and physical examination.

Primary Outcome Measures. An independent evaluator (IE), blind to study condition, will administer the diagnostic assessments and clinician-rated symptom scales. All IEs will be experienced clinicians who will have undergone specific assessment training. We will adopt the specific guidelines for completing the scales based on experiences in previous trials of social anxiety disorder and other related disorders conduced by our group [3]. Specifically, the scales used in this study have specific, carefully defined, anchors. Furthermore, Dr. Smits, who will lead the quality assurance/quality control effort for this trial, will periodically review assessment recordings and meet with clinical assessors to address potential drift.

<u>The Liebowitz Social Anxiety Scale (LSAS [41])</u>. This is a 24-item scale that provides separate scores for fear and avoidance in social and performance situations; it is widely used in treatment studies of SAD. The instrument shows very good psychometric properties (Heimberg et al., 1999; Safren et al., 1999). The LSAS will be administered by the IE at baseline, at each treatment session, at post-treatment, and the 1- and 3-month follow-up assessment

<u>Social Phobic Disorders Severity and Change Form (SPD-SC Form [42])</u>. This is an expansion and adaptation of the Clinical Global Impression Scale (CGI) by Guy [43] to social anxiety disorder. Similar to the original CGI scale, the SPD-SC Form is rated on a 7-point scale to indicate severity and improvement. We chose this scale over the original CGI scale because it provides a more detailed analysis of psychological functioning for individuals with SAD. Furthermore, other studies (e.g., Heimberg et al., 1998) used the SPD-SC Form, but not the CGI. The SPD-SC Form will be administered by the IE at baseline, at each treatment session, at post-treatment, and the 1- and 3-month follow-up assessment.

Secondary Outcome Measures

<u>Montgomery Asberg Depression Rating Scale (MADRS [44])</u>. The MADRS is designed to measure the overall severity of depressive symptoms and has demonstrated good reliability, specificity for depressive compared to anxiety symptomatology, and sensitivity to change with treatment. The MADRS will be used to

assess depression as a potential treatment moderator. The MADRS will be administered by IEs at baseline, weekly during treatment, at post-treatment, and the 1- and 3-month follow-up assessment.

Quality of Life Enjoyment and Satisfaction Questionnaire (Q-LES-Q;[45]). This questionnaire rates 16 aspects of quality of life, including physical health, mood, activities of daily living, and overall life satisfaction.). The Q-LES-Q will be used to examine changes in quality of life with treatment. It will be self-administered at pre-treatment, post-treatment, and the 1- and 3-month follow-up assessment.

In-Session Fear Ratings. Participants will provide fear ratings at the beginning of an exposure exercise (i.e., Beginning Fear) and just prior to the conclusion of an exposure exercise (i.e., End Fear). In addition, they will indicate their highest level of fear experienced during exposure after the exercise (i.e., Peak Fear). Fear ratings will be assessed using the subjective units of distress scale (SUDs; [46]), which ranges from 0 to 100 (0=no fear, relaxed; 25=mild fear, able to cope; 50=moderate fear, trouble concentrating; 75=severe fear, thoughts of leaving; 100=very severe fear, worst ever experienced). The procedures for collecting fear ratings were similar to that in previous social anxiety disorder treatment studies from our group and other groups [2, 4, 47-49]. Specifically, during the first session, therapists will introduce patients to the SUDs scale as they work together to develop a fear and avoidance hierarchy. Attention will be given to the anchors such that patients can distinguish the different levels along the scale. Accordingly, by the time patients initiate exposure practice (i.e., session 2), they will have had had ample practice using the scale. We will use a fear rating at the end of exposure of ≤ 40 as an index of exposure success (see Table 2 and section 3C.4f).

Moderator Variables

Demographic Variables. Participants will be asked to provide standard demographic information (age, sex, race/ethnicity, level of education, cohabitation status) using forms employed in previous studies.

Clinical Characteristics. Baseline psychiatric functioning will be assessed by clinician-rated measures. This domain comprises clinical severity (as assessed by the Social Phobic Disorders Severity Form; SPD-SC; Liebowitz [42], depressive symptom severity (as assessed by the MADRS [44]), Axis I comorbidity (i.e., number of comorbid Axis I disorders as assessed by the SCID) as well as history of antidepressant and other psychotropic use.

Personality Traits. At the baseline session, participants will complete the 60-item NEO-Five-Factor Inventory (NEO-FFI [50]), which is a psychometrically-sound measure of the five traits from the Five-Factor model of personality: agreeableness, conscientiousness, extraversion, neuroticism, and openness.

Measures of Treatment Integrity and Acceptance

Credibility and Expectancy. The Credibility/Expectancy Questionnaire (CEQ) is a widely used 6-item measure assesses treatment credibility and expectancy. It will be self-administered after the first treatment session.

Table 3. Assessment Schedule

Measures	Screen/Baseline	Each Session	Posttreatment	1-M Follow-up	3-M Follow-up
Screening					
Eligibility Screen	X				
Lab Testing	X				
SCID	X				
Primary Outcomes					
LSAS	X	X	X	X	X
SPD-SC Form	X	X	X	X	X
Secondary Outcomes					
MADRS	X	X	X	X	X
Q-LES-Q	X		X	X	X
Exposure Success					
SUDS		X			
Moderators					
Demographics	X				
Clinical Characteristics	X				
Personality Traits	X				
Treatment Integrity and Acceptance					
Credibility and Expectancy		X			
Adherence		X			
Safety		X			

Adherence. Patient adherence to each intervention will be assessed as the number of total sessions attended.

Safety. Patients will be queried at each visit regarding the presence of adverse effects associated with the study medication. Review of medical history, physical examination, and laboratory tests will be performed at admission, and vital signs measured at every visit. Patients with clinically significant abnormalities in vital signs (e.g., systolic blood pressure >150 mm Hg or diastolic blood pressure >100 mm Hg) at baseline or for two consecutive evaluations will be excluded from further study participation and referred for appropriate clinical management.

3C.6: Data Analysis.

3C.6a: Overview. Data collection, coordination, and integrity at all 3 sites will be supervised by the data management team, consisting of the three PIs (Drs. Pollack, Hofmann, and Smits) and the project biostatistician (Dr. Rosenfield; see data management plan). Measures will be completed using a tablet computer employing questionnaire management software (Qualtrics). We will assess the equivalence of the treatment groups on key baseline variables (demographics and psychological variables); variables on which the groups differ will be used as covariates in the final analyses. We will then examine missing data patterns, dropout rates (see below), and distributional properties of measures and use transformations to improve distributions if necessary. We will use multilevel modeling (MLM) to evaluate the effect of DCS on our continuous outcome measures (LSAS, SPD-SC severity, MADRS, QLES-Q) and use GLMM (MLM with a logistic linking function) to examine DCS's effect on the dichotomous outcomes ("response" and "remission" see 3.C.8b for the definition).

MLM is the recommended method for analyzing longitudinal psychiatric data [51], easily accommodates missing data, and allows inclusion of all subjects in the analysis even if they drop out. In our MLM analyses, the repeated assessments over time will be nested within individuals, which will be nested within their treatment cohort, thereby appropriately accounting for correlated scores within cohorts. We will use a piecewise growth curve model, separately modeling change over time during treatment and during follow-up. Although our prior large DCS study [3] found each "piece" of the growth curve to be linear, we will test for quadratic trends and include them if significant. Our "time" variable in these models will be coded as "assessment week" and will reflect the number of weeks since baseline. Using this model, we can test differences between tailored DCS administration and the 3 comparison groups (DCS-pre, DCS-post, PBO) by including 3 dummy coded variables as predictors of the growth curve parameters. Each dummy variable will contrast tailored post-DCS to each of the other 3 conditions. All models will include relevant control variables (e.g., gender, education, initial severity, etc.). Tests of group differences (the contrasts between tailored DCS and each of the other conditions) at post and at each follow-up will be conducted by centering the "assessment week" variable in our MLM model at the appropriate time point for which we are testing group differences (e.g., "assessment week" will be centered at post to test for groups differences at posttreatment [52]). Secondarily, slope differences between conditions will also be assessed.

3C.6b: Definition of Treatment Response and Remission.

The definition of treatment "response" is based on the SPD-SC and is defined by an overall SPD-SC change score of 2 (much improved) or 1 (very much improved) as compared to the pre-treatment assessment. "Remission" is defined as an SPD-SC of 2 or 1 and an LSAS total score of < 30. This LSAS cutoff score is supported by a study of 364 patients that used receiver-operating characteristics in diagnosing SAD [53]. This score has been generally adopted as representing the boundary between remitted and symptomatic patients [54]. Please note that, consistent with the general aims of an R34, our goal is to determine effect size estimates for these gold-standard outcomes and whether a Stage II study is warranted, rather than to detect differences on our dichotomous outcomes (response and remission), which would require a very large sample size.

3C.6c: Tests of Specific Aims.

Aim 1: A significant difference between tailored post DCS and each of the comparison conditions will be indicated if the dummy variable contrasting tailored DCS to the relevant comparison condition is a significant predictor of the intercept. We will alternately "center" the "assessment week" variable at either posttreatment, 1-mo FU, or 3-mo FU to test for significant differences between conditions at each of these time points [52].

Aim 2 (Exploratory investigation of potential moderators): We will examine numerous possible moderators (demographic, clinical, and personality variables) of the potential superiority of tailored DCS over the comparison conditions (and, secondarily, of the superiority of pre-and post-DCS administration over PBO). We will use the Fournier approach [55, 56] to identify important moderators. This approach uses an algorithmic method to select significant predictors/moderators within each group of potential moderators (the groups being demographics, clinical characteristics, personality variables) and then combines these selected moderators in a final overall analysis, which identifies moderators that are significant over and above the other potential moderators. This approach strikes a balance between testing each moderator separately (which substantially increases Type I error due to the multiple tests) and testing all moderators simultaneously (which may substantially increase the likelihood of Type II error). We have successfully used this approach to examining the moderators of pre-session administration of DCS [56].

3C.6d: Missing Data. Following Enders [57], we will use pattern mixture modeling to assess the effect of missing data. We will rerun our analyses coding for various missing data patterns (no missing data, sporadic missing, dropouts, etc.) to determine 1) if missingness impacts our findings and 2) how the differences

between treatment conditions depends on the missing data pattern.

3C.6e: Power Analysis. Because we will have a sample size of 156, this application is not powered to detect small differences between treatment conditions, nor is it powered to detect differences on our dichotomous outcomes. However, consistent with the aims of a R34, our primary goal is determining 1) the effectiveness of tailored DCS-post and 2) whether a Stage II study is warranted. Thus, we performed power analyses only for our primary outcomes, which were continuous measures of LSAS and SPD-SC.

MLM allows the inclusion of all subjects with at least one data point, regardless of missing data and regardless of whether they drop out. Thus, we based our power analysis on 156 participants and conservatively assumed that, on average, we will obtain 5 out of the 7 total assessments from each subject. The power analyses were calculated using the program PinT 2.12 (Power in Two Level Models [58]).

Aim 1: We used our similarly sized DCS trial [3] to estimate the variances and covariances required by PinT, and calculated detectable effect sizes for both LSAS and SPD-SC. PinT indicates that we would have a power of .80 to detect an effect size as low as d=.296, between a small (d=.20) and a medium (d=.50) effect size. This translates into a difference of 6.59 points on the LSAS and .42 points on the SPD-SC (at post, or 1-mo FU, or the 3-mo FU).

Aim 2: For the Fournier moderator analysis, we assumed five simultaneous moderators in each group, and included their 15 interactions with the 3 dummy variable contrasts. Moderators were modeled as predictors of both the intercept and slope. Again using our DCS study to calculate the variances and covariances required by PiNT, we found that, with .80 power, we could detect an effect size of d=.466, slightly smaller than a medium effect size.

PROTECTION OF HUMAN SUBJECTS

Risks to the Subjects.

Human Subjects Involvement and Characteristics. Participants will be 156 adults with generalized SAD. All interested participants meeting entrance criteria will be offered the opportunity to participate. All participants will undergo psychiatric and medical screening and baseline assessment prior to starting a 5-session cognitive-behavioral treatment protocol. Participants will be enrolled into a 5-session CBT protocol and randomly assigned to 1) Tailored post-session DCS: pre-session PBO and selective post-session DCS administration during sessions 2-5; (2) Pre-session DCS: pre-session administration of 50 mg of DCS and post-session administration of pill placebo (PBO) (during sessions 2-5); (3) Placebo; pre- and post-session session administration of PBO (during sessions 2-5) or (4) Non-tailored post-session DCS: pre-session PBO and post-session DCS administration (during sessions 2-5; see Table 1.). During treatment sessions 2-5, participants will complete exposure exercises. They will be also asked to complete interviews and self-report questionnaires about anxiety and mood symptoms as well as provide blood and urine samples (see Table 3 for the assessment schedule)

Sources of Research Material. Data will be obtained from patients recruited specifically for this protocol. Demographic and diagnostic data, monitoring clinician assessments and ratings, and patient self-report questionnaires will be collected and used for information related to study participation and treatment efficacy. Medical evaluations, including blood and urine samples, blood pressure and pulse rate will be used for information related to study participation and treatment safety. Subjects will be recruited from patients that present for treatment at the Center for Anxiety and Traumatic Stress Disorders at Rush University Medical Center, the Center for Anxiety and Related Disorders at Boston University, and the Institute for Mental Health Research at The University of Texas at Austin. Patients will also be solicited through the media and other clinic facilities and programs. Referrals are made by medical and mental health practitioners in the area as well as self-referrals by affected individuals. Individuals who contact the clinic will be phone screened for general medical and diagnostic treatment eligibility and interest in research participation.

Potential Risks. This study is designed to provide dose-timing information on the relative efficacy of the addition of DCS to exposure based CBT for SAD compared to the addition of placebo. In addition to providing well-monitored clinical interventions with an effective psychosocial treatment for SAD, and the possibility that augmentation with DCS may offer additional benefit to participants in this study, information derived from the study may improve the treatment of future patients with SAD. The potential side effects associated with the administration of DCS include drowsiness, headache, prolonged or momentary dizziness, seizures, confusion, hallucinations, weakness, coma, rash, vitamin B_{12} deficiency and/or folate deficiency (both of which may cause weakness and anemia (low blood counts)), liver enzymes increases (which could cause weakness or bleeding), and shaking. However, these side effects are most commonly related to doses greater than 500mg/day, which is at least ten times what is administered in this study. The use of DCS at doses of 50 mg (as in this study) does not appear to be associated with significant emergent adverse effects.

Some participants may feel uncomfortable about having treatment sessions audiotaped and reviewed by others (necessary for therapist supervision and treatment adherence). Additionally, clients may experience some disruption of daily activities due to scheduling of treatment sessions. Some treatment procedures, particularly the in vivo exposures are likely to provoke some discomfort. Participants will be informed about these risks and told that they may withdraw from the study at any time and may refuse to complete any treatment procedures they find too uncomfortable.

Protection of Human Subjects from Research Risk.

Recruitment and Informed Consent. Participants will be obtained through: (a) referral from area medical and mental health professionals, (b) community outreach, and (c) advertisements placed in local media. Any subjects meeting the entrance inclusion criteria will be provided the opportunity to participate in this study. Specific procedures are in place to maximize our aggregation of a racially and ethnically diverse sample. Community mental health centers and medical clinics will be informed of the project and referrals will be encouraged from primary care physicians and clinics. If necessary, special attention will be given to churches and other social groups with high minority representation to ensure adequate accrual of racially/ethnically diverse subjects.

In accordance with HIPAA regulations, written informed consent will be obtained from each participant after a thorough explanation of procedures by a project staff person and the participant will be given the opportunity to ask and receive answers to questions. Participants will be informed of the nature of the investigation, the types of assessments and interventions involved, alternative interventions, and the potential risks involved in

participation. In addition, an explanation of how information related to their case will be handled, including data management and plans to publish data in group format without identifying information, will be presented. The Institutional Review Board will have approved the protocol, consent, and HIPAA authorization forms prior to the initiation of the study.

Adequacy of Protection Against Risks. We developed and followed strict safety guidelines during previous and pilot studies that will be applicable to the proposed study:

1. Careful screening to identify patients whose risk for potential adverse outcomes is elevated were they to participate in the proposed research. Such patients including actively suicidal patients would be excluded from study participation and provided with appropriate clinical treatment.
2. All patients will meet regularly with a physician to monitor the emergence of adverse effects, as well as with clinicians experienced in the assessment and treatment of patients with social anxiety disorder. The treatment program formalizes assessment and monitoring of symptoms and adverse effects.
3. The study exclusion criteria include comorbid psychiatric disorders that may complicate the treatment process.
4. Patients are tested for alcohol prior to the administration of study medication in order to reduce the possibility of an adverse interaction.
5. In all phases of the research, participants will be instructed to contact study personnel at any time in the event of worsening of symptoms or relapse. Participants whose clinical condition has deteriorated will be removed from the study and given appropriate clinical care. This will be operationalized as all patients who have an increase in the Social Phobic Disorders Change Form (SPD-C) of greater than 5 (more than minimally worse) for two consecutive visits and any patient who becomes suicidal will be removed from the study protocol and treated clinically. All sites have trained clinical staff available by pager at all times to handle emergencies.
6. Participants failing to benefit from the study interventions will be provided with appropriate clinical care. Participants who begin the intervention and experience adverse outcomes sufficient to require removal from the study will receive open clinical care. The exact nature of "appropriate clinical care" will be determined by the judgment of clinicians familiar with the specific participant in collaboration with the subject and may include CBT, other psychotherapy, or referral for psychiatric treatment. Following completion of the study protocol, we assist participants in finding appropriate follow up care if needed.
7. The DSMB will oversee safety and other related issues pertinent to the ongoing study. Twenty-hour/day emergency coverage with a study clinician will be available at each of the 3 sites. Patients are provided with cards with the emergency contact number. In the event of an emergency, the clinician will determine the necessary clinical intervention and provide and coordinate appropriate care.
8. As in any type of treatment or clinical research program, participants' confidentiality must be carefully guarded and respected. All data with identifying information will be stored in locked files or password-protected computer files. Data being analyzed will be identified by subject codes, and identifying information will be removed. The identity of participants will not be revealed in the presentation or publication of any results from the project. All assistants and others working on the project will be educated about the importance of strictly respecting participants' rights to confidentiality and will have completed training concerning proper practice in accordance with the Healthcare Information Portability and Accountability Act (HIPAA) regulations.
9. Recording of IE clinical interviews will be a required procedure. The purpose of the recording will be explained, confidentiality will be respected, and both informed consent and authorization for recording will be obtained as per requirements put forth by HIPAA. Digital recordings will be stored and moved between sites using a secure, password-protected and HIPAA-compliant website. Recordings will be stored under lock and key for use in further ratings and maintained until five years after the publication of study results in line with the guidelines of the American Psychological Association.

Potential Benefits of Proposed Research to the Subjects and Importance of Knowledge to be Gained

Subjects who participate in this study may benefit from the close monitoring and interventions provided to them. These potential benefits are provided without charge. Information provided as part of the treatment program may also help participants better understand the relationship between behaviors, thoughts and anxiety, and through understanding their disorder, maintain improvement over the long term. The primary risks to the patient are medication-related side effects, which based on review of the literature and experience to date appear to be minimal, and discomfort associated with the assessments. Study personnel will be monitoring the patients’ clinical condition carefully and will withdraw patients from the study if their clinical

condition warrants withdrawal. This study promises to provide important information about the relative efficacy and safety of a novel treatment strategy to improve outcome for patients with social anxiety disorder. The potential benefits of this study to patients suffering from social anxiety disorder justify the risks involved.

Data Monitoring and Management Procedures

Integrity of Diagnostic Assessment. All SCID interviews will be conducted by certified clinicians who undergo strict training procedures under the supervision of the PIs (clinical researchers with many years of diagnostic experience). Training will include review of video recordings and participation in live SCID interviews. The certification procedure requires the trainee to (1) view 3-4 videotaped and live SCIDadministrations by senior interviewers with the comparison of the trainees' ratings to that of the senior interviewer, and (2) administer 6-10 SCID interviews in the presence of the senior interviewer with the requirement that the trainees' diagnosis match that of the senior interviewer on at least 4 of 5 consecutive administrations. In addition to the training, study staff will hold weekly meetings during which all interviews conducted that week are discussed in the presence of the PIs.

Reliability of Diagnostic and Independent Efficacy Ratings. The IEs for the proposed study will be Ph.D. or Master's level diagnosticians who have previous research experience with structured interviewing. A two-level system will be used to maintain the reliability of diagnoses and of other clinical ratings in the study. At least 10% of all intakes will be interviewed by two independent clinicians. Furthermore, all clinical assessments will be digitally recorded. Each month, an independent evaluator (IE) will listen to an assessment performed by another IE associated with the study and independently complete the assessment instruments. These duplicate ratings will be used both to calculate kappa coefficients and for supervision. Differences between raters will be discussed during supervision to identify reasons for disagreement and improve inter-rater reliability. These procedures will help to ensure that IEs refine their diagnostic skills and will also establish common guidelines for ongoing use in diagnostic decision-making. Inter-rater agreement will be assessed via evaluation of digital recordings of diagnostic interviews (as described above). Inter-rater reliability (kappa) will be calculated for all anxiety disorders and MDD. Dr. Powers at the UT Austin site will be responsible for overseeing the diagnostic and efficacy quality assurance efforts for the study.

Procedures for Data Management. Our general policy for data management is that research assistants copy all data files and these files are brought to the PIs on a bi-weekly basis. Data forms and accompanying narrative summaries will undergo a systematic and rigorous editing process prior being keyed into the database. The research assistants routinely evaluate the data and discuss any problems and questions with the study staff at the weekly team meetings. Accuracy of data entry will be ensured by a standard double-entry procedure. Data management formal reports on record status across the three following domains will be employed: entered, verified, and edited. These reports of data records will be evaluated 1 time a month during the final team meeting of the month. To help ensure data protection, backup copies, automatically generated by our computer systems, will be available. Additionally, our hard copy record systems, as described previously, will be maintained in fire-resistant locked cabinets.

This study will utilize a web page-internet data collection and management system used in previous work. All data for the current study including demographic information, laboratory values, and participant and clinician rated measures will be directly entered into an electronic case report form (eCRF). The eCRF will be entered into web pages using a dedicated personal computer at each respective site. The web pages will be accessed at a central site using a standard Internet browser.

The eCRF will consist of a series of separate web pages for study personnel and participants. A series of passwords will be programmed to ensure that participants are unable to access pages reserved for study personnel. The eCRF will be constructed so that all requested information must be entered into each page in the fields provided, or the system will not permit access to the next page. The system is designed so that only completed eCRFs can be transmitted. If information for a field is either not available or not applicable, the system will require that it be documented as such in the eCRF. Field parameters will be specified such that suspect values are either disallowed or flagged for the immediate attention of the study coordinators and Principal investigators.

The completed eCRF will be transmitted to the central site using encryption code, at the completion of the study visit. In addition to transmitting the completed eCRF to the central site, at each visit a hard copy of the eCRF will be printed and promptly reviewed, signed, and dated by the investigator for clinician rated measures and by the participant for participant rated measures. Data will not be transmitted until reviewed by the participant, investigator and research assistant for completeness and accuracy. A print out of the data will then

be made, authenticated (with signature and date) by the investigator and participant and kept in the participant's study file.

Confidentiality is assured by a number of factors. Most importantly, participants will be identified on the eCRF only by participant number, visit number, and date of visit, assuring confidentiality of the anonymized data on the web. By recording the study data in this manner, the information can be considered 'de-identified,' and therefore, compliant with the Standards for Privacy of Individually Identifiable Health Information ("Privacy Rule") of the Health Insurance Portability Act of 1996 ("HIPAA"). Additional measures to ensure the confidentially of study data include the following: A dedicated personal computer at each investigational site will permit the electronic authentication/signature of all information and data collected during the study. When data are submitted, the user id, password, date, time, and IP address of the computer are logged. As a result, the number of locations from which the database can be accessed will be limited, effectively restricting access to individual computers. Access to the dedicated personal computer at the study site will be restricted to participants, investigators and staff involved in the study. Each user of the system will be assigned a unique user-id. Each user-id will be associated with a subset of participants. Thus, project staff will only be able to access the records of participants for whom they are responsible and for those individuals registered in the study at that site (to allow for cross coverage of participants when necessary). Data will be accessed by participant number, visit number, and specified form of interest. Participants will have access only to the current visit, and only to the subset of forms that they will be filling out. As a result, participants will require the assistance of project staff member to access other aspects of their record.

The security of the database is maintained and any changes or modifications to the eCRF record rigorously documented. The current record is modified using the web page-internet technology each time an eCRF data file is accessed. Every access of an eCRF will be logged in a separate archival file, which will permit PIs to track who made the data changes, the dates and times of the data changes, and which data fields were changed. In addition, the technology permits the recovery of the data entered previous to any given change. It is important to note that this recording is invisible (and inaccessible) to users at the study site (the end user), and will be available only to the Data Manager and the PIs. The physical security of the data will be maintained in a number of ways. All data will be maintained on the mainframe computers the respective sites. As a result, the data will be fully backed up and fire protected. Backups are performed in real time, and the back up tapes are stored in a fire protected setting in an off-site location. All forms will be printable, if necessary. At the conclusion of the study, the database will be permanently archived at each respective site.

Data Safety and Monitoring Plan

This Human Subjects Research meets the definition of a clinical trial. The following procedures will be followed, in compliance with NIH requirements, to ensure the safety of study participants and the validity and integrity of data.

Functions of the Data and Safety Monitoring Board (DSMB). A Data and Safety Monitoring Board (DSMB) will be created to ensure that the safety of study subjects is protected and that the scientific goals of the study are being met. To support those purposes, the DSMB will review any proposed amendments to the study protocol, perform expedited monitoring of all serious adverse events, perform ongoing monitoring of drop-outs and non-serious adverse events, determine whether study procedures should be changed or the study should be halted for reasons related to the safety of study subjects, and perform periodic review of the completeness and validity of data to be used for analysis of safety and efficacy. The DSMB will also ensure subject privacy and research data confidentiality.

Membership of the DSMB. To fulfill its mission of ensuring the safety and integrity of the study, it is necessary that the DSMB be comprised of members who possess a high degree of competence and experience, as well as the ability to function independently of all other parties involved in the study. The DSMB members should function free of the career and financial interests of its members. The DSMB will consist of three members with experience in conducting clinical trials for psychiatric disorders, expertise in biostatistics, and a thorough knowledge of clinical trial ethics and human subject protection issues (Drs. Murray Stein, Gail Steketee, and Sabine Wilhelm).

Functional Organization of the DSMB. The Chairperson of the DSMB will communicate by e-mail and telephone conference with the other members. Communication pertaining to review of serious adverse events (SAEs) will occur within a week of receiving any new SAE report. Reporting and communication about other matters will occur on a yearly basis, for the duration of the study. Decisions of the DSMB will be made based on a majority vote of the members.

Monitoring of Safety Data by the DSMB.

Unblinded Reporting. Safety information for this study will be reported to the DSMB in an unblinded manner. A statistical penalty will not be assessed for the ongoing unblinded review of safety by the DSMB. Unblinded data will not be released to the investigators unless necessary for safety reasons.

Range of Safety Reporting to the DSMB. It is considered necessary for the purpose of monitoring the safety of the study that the DSMB review not only adverse events (AEs) and serious adverse events (SAEs), but also other data that may reflect differences in safety between groups. This includes treatment retention rates and reasons for dropout.

Serious Adverse Events. Expedited review will occur for all events meeting the FDA definition of Serious Adverse Events (SAEs; i.e., any fatal event, immediately life-threatening event, permanently or substantially disabling event, event requiring or prolonging inpatient hospitalization, or any congenital anomaly). This also includes any event that a study investigator or the DSMB judges to impose a significant hazard, contraindication, side effect, or precaution. For purposes of this study, <u>all</u> SAEs will be required to be reported to the DSMB, regardless of any judgment of their relatedness to the study. All relevant information will be reported to the DSMB for each SAE including information about the event and its outcome, study condition, concomitant medications, the subject's medical history and current conditions, and all relevant laboratory data. Notification by e-mail and FAX transmittal of all related study forms shall be made to the DSMB within 2 days of the occurrence of any SAE. Information will be reviewed and a determination made of whether there was any possible relevance to the study interventions. Additional reporting to local IRBs will be done within 24 hours of the SAE; reporting to NIH will be made according to their respective regulations governing SAE reporting.

Non-Serious Adverse Events. At yearly intervals during the course of the study and then again at its completion, the DSMB will be provided with unblinded summaries of the numbers and rates of adverse events by treatment group. These reports will include types of events, severity, and treatment phase. Data on individual non-serious adverse events is not expected to be needed for this review.

Other Safety-Related Reports. At yearly intervals throughout the course of the study, the DSMB will also receive unblinded summary reports of treatment retention and reasons for dropout, by treatment arm and study phase.

Study Stopping Rules. If at any time during the course of the study, the DSMB judges that risk to subjects outweighs the potential benefits, the DSMB shall have the discretion and responsibility to recommend that the study be terminated.

<u>D6e. Monitoring of Data Quality by the DSMB.</u> At least on an annual basis during the course of the study, the DSMB will receive a report on data quality and completeness. At a minimum, this will include an overview of the progress of participant intake and retention; summary reports describing participant compliance with visits, evaluations, and dosing as described in the protocol; and a summary of the completeness and quality of key data elements needed to characterize participants, their dosing, and their primary and secondary outcomes. These reports will be used by the DSMB to evaluate the capacity of the data capture and processing to support scientifically valid analyses.

Annual DSMB Report to NIMH. Annually during the course of the study the DSMB will prepare a summary report of its findings regarding safety and quality based on data received to that point in the study. This report will include a summary of all safety findings, as well as an assessment of protocol compliance and data quality. Any recommendations to improve participant safety, protocol adherence, or data quality will be made in the annual DSMB report. A copy of the annual DSMB report will be sent to the local IRBs along with the annual renewal report.

REFERENCES

1. Litz BT, Salters-Pedneault K, Steenkamp MM, et al. A randomized placebo-controlled trial of d-cycloserine and exposure therapy for posttraumatic stress disorder. J Psychiatr Res. 2012;46:1184-1190.
2. Smits JA, Rosenfield D, Otto MW, et al. D-cycloserine enhancement of exposure therapy for social anxiety disorder depends on the success of exposure sessions. J Psychiatr Res. in press.
3. Hofmann SG, Smits JA, Rosenfield D, et al. D-Cycloserine as an augmentation strategy with cognitive-behavioral therapy for social anxiety disorder. Am J Psychiatry. 2013;170:751-58.
4. Smits JA, Rosenfield D, Otto MW, et al. D-Cycloserine Enhancement of Fear Extinction is Specific to Successful Exposure Sessions: Evidence from the Treatment of Height Phobia. Biol Psychiatry. 2013;73:1054-58.
5. Tart CD, Handelsman PR, Deboer LB, et al. Augmentation of exposure therapy with post-session administration of D-cycloserine. J Psychiatr Res. 2013;47:168-74.
6. Smits JA, Rosenfield D, Davis ML, et al. Yohimbine enhancement of exposure therapy for social anxiety disorder: A randomized controlled trial. Under Review.
7. Hofmann SG, Smits JA. Cognitive-behavioral therapy for adult anxiety disorders: a meta-analysis of randomized placebo-controlled trials. J Clin Psychiatry. 2008;69(4):621-32.
8. Davidson JR, Foa EB, Huppert JD, et al. Fluoxetine, comprehensive cognitive behavioral therapy, and placebo in generalized social phobia. Arch Gen Psychiatry. 2004;61:1005-13.
9. Barlow DH, Gorman, JM, Shear, MK, Woods, SW. Cognitive-behavioral therapy, imiprimine, or their combination for panic disorder: A randomized controlled trial. JAMA. 2000;283:2529-36.
10. Foa EB, Liebowitz MR, Kozak MJ, et al. Randomized, placebo-controlled trial of exposure and ritual prevention, clomipramine, and their combination in the treatment of obsessive-compulsive disorder. Am J Psychiatry. 2005;162:151-61.
11. Davis M, Ressler K, Rothbaum BO, Richardson R. Effects of D-cycloserine on extinction: translation from preclinical to clinical work. Biol Psychiatry. 2006;60:369-75.
12. Otto MW, Tolin DF, Simon NM, et al. Efficacy of D-cycloserine for enhancing response to cognitive-behavior therapy for panic disorder. Biol Psychiatry. 2010;67:365-70.
13. Hofmann SG, Meuret AE, Smits JA, et al. Augmentation of exposure therapy with D-cycloserine for social anxiety disorder. Arch Gen Psychiatry. 2006;63:298-304.
14. Ressler KJ, Rothbaum BO, Tannenbaum L, et al. Cognitive enhancers as adjuncts to psychotherapy: use of D-cycloserine in phobic individuals to facilitate extinction of fear. Arch Gen Psychiatry. 2004;61:1136-44.
15. Guastella AJ, Richardson, R, Lovibond, PF, Rapee, RM, Gaston, JE, Mitchell, P, Dadds, MR. . A randomized controlled trial of the effect of d-cycloserine on enhancement of exposure therapy for social anxiety disorder. Biol Psychiatry. 2008;63:544-549.
16. Wilhelm S, Buhlmann U, Tolin DF, et al. Augmentation of behavior therapy with D-cycloserine for obsessive-compulsive disorder. Am J Psychiatry. 2008;165:335-41.
17. Guastella AJ, Lovibond PF, Dadds MR, Mitchell P, Richardson R. A randomized controlled trial of the effect of D-cycloserine on extinction and fear conditioning in humans. Behav Res Ther. 2007;45:663-72.
18. Weber M, Hart J, Richardson R. Effects of D-cycloserine on extinction of learned fear to an olfactory cue. Neurobiol Learn Mem. May 2007;87(4):476-82.
19. Bouton ME, Vurbic D, Woods AM. D-cycloserine facilitates context-specific fear extinction learning. Neurobiol Learn Mem. 2008;90:504-10.
20. Ledgerwood L, Richardson R, Cranney J. Effects of D-cycloserine on extinction of conditioned freezing. Behav Neurosci. 2003;117:341-9.
21. Parnas AS, Weber M, Richardson R. Effects of multiple exposures to D-cycloserine on extinction of conditioned fear in rats. Neurobiol Learn Mem. 2005;83:224-31.
22. Hofmann SG, Smits JA, Asnaani A, Gutner CA, Otto MW. Cognitive enhancers for anxiety disorders. Pharmacol Biochem Behav. 2011;99:275-84.
23. Kessler RC, Chiu WT, Demler O, Merikangas KR, Walters EE. Prevalence, severity, and comorbidity of 12-month DSM-IV disorders in the National Comorbidity Survey Replication. Arch Gen Psychiatry. 2005;62:617-27.

24. Kessler RC, Berglund P, Demler O, Jin R, Merikangas KR, Walters EE. Lifetime prevalence and age-of-onset distributions of DSM-IV disorders in the National Comorbidity Survey Replication. Arch Gen Psychiatry. 2005;62:593-602.
25. Stein MB, Roy-Byrne PP, Craske MG, et al. Functional impact and health utility of anxiety disorders in primary care outpatients. Med Care. 2005;43:1164-70.
26. Greenberg PE, Sisitsky T, Kessler RC, et al. The economic burden of anxiety disorders in the 1990s. J Clin Psychiatry. Jul 1999;60(7):427-35.
27. Otto MW, Smits JA, Reese HE. Cognitive-behavioral therapy for the treatment of anxiety disorders. J Clin Psychiatry. 2004;65 Suppl 5:34-41.
28. Anderson KC, Insel TR. The promise of extinction research for the prevention and treatment of anxiety disorders. Biol Psychiatry. 2006;60:319-21.
29. Falls WA, J. MM, Davis M. Extinction of fear-potentiated startle: Blockade by infusion of an NMDA antagonist into the amygdala. J Neurosci. 1992;12:854-63.
30. Walker DL, Ressler KJ, Lu KT, Davis M. Facilitation of conditioned fear extinction by systemic administration or intra-amygdala infusions of D-cycloserine as assessed with fear-potentiated startle in rats. J Neurosci. 15 2002;22:2343-51.
31. Guastella AJ, Dadds MR, Lovibond PF, Mitchell P, Richardson R. A randomized controlled trial of the effect of D-cycloserine on exposure therapy for spider fear. J Psychiatr Res. 2007;41:466-71.
32. Santini. Consolidation of extinction learning involves transfer from NMDA-independent to NMDA-dependent memory. J Neurosci. 2001;21:9009-17.
33. Woods AM, Bouton ME. D-cycloserine facilitates extinction but does not eliminate renewal of the conditioned emotional response. Behav Neurosci. 2006;120:1159-62.
34. Lee JL, Milton AL, Everitt BJ. Reconsolidation and extinction of conditioned fear: inhibition and potentiation. J Neurosci. 2006;26:10051-56.
35. Przybyslawski J, Sara SJ. Reconsolidation of memory after its reactivation. Behav Brain Res. 1997;84:241-46.
36. Suzuki A, Josselyn SA, Frankland PW, Masushige S, Silva AJ, Kida S. Memory reconsolidation and extinction have distinct temporal and biochemical signatures. J Neurosci. 2004;24:4787-95.
37. Torras-Garcia M, Lelong J, Tronel S, Sara SJ. Reconsolidation after remembering an odor-reward association requires NMDA receptors. Learn Mem. 2005;12:18-22.
38. Insel T, Cuthbert B, Garvey M, et al. Research domain criteria (RDoC): toward a new classification framework for research on mental disorders. Am J Psychiatry. 2010;167:748-51.
39. D'Souza DC, Gil R, Cassello K, et al. IV glycine and oral D-cycloserine effects on plasma and CSF amino acids in healthy humans. Biol Psychiatry. 2000;47:450-62.
40. van Berckel BN, Lipsch C, Timp S, et al. Behavioral and neuroendocrine effects of the partial NMDA agonist D-cycloserine in healthy subjects. Neuropsychopharmacology. 1997;16:317-24.
41. Liebowitz MR. Social phobia. Modern problems in pharmacopsychiatry. 1987;22:141-73.
42. Liebowitz MR, Schneier F, Campeas R, et al. Phenelzine vs atenolol in social phobia. A placebo-controlled comparison. Arch Gen Psychiatry. 1992;49:290-300.
43. Guy W. Clinical global impression. Manual for the ECDEU Assessment Battery. 1970.
44. Montgomery SA, Asberg M. A new depression scale designed to be sensitive to change. Br J Psychiatry. 1979;134:382-9.
45. Endicott J, Nee J, Harrison W, Blumenthal R. Quality of Life Enjoyment and Satisfaction Questionnaire: a new measure. Psychopharmacol Bull. 1993;29:321-26.
46. Wolpe, J. Psychotherapy by reciprocal inhibition. Stanford University Press; 1958.
47. Smits JA, Powers MB, Buxkamper R, Telch MJ. The efficacy of videotape feedback for enhancing the effects of exposure-based treatment for social anxiety disorder: a controlled investigation. Behav Res Ther. 2006;44:1773-85.
48. Smits JA, Rosenfield D, McDonald R, Telch MJ. Cognitive mechanisms of social anxiety reduction: an examination of specificity and temporality. J Consult Clin Psychol. 2006;74(:1203-12.
49. Hayes SA, Hope DA, Heimberg RG. The pattern of subjective anxiety during in-session exposures over the course of cognitive-behavioral therapy for clients with social anxiety disorder. Behav Ther. 2008;39:286-99.
50. Costa PT, McCrae RR. Revised NEO Personality Inventory (NEO-PI-R) and NEO Five-Factor Inventory (NEO-FFI) manual. Odessa, FL: Psychological Assessment Resources; 1992.

51. Hamer RM, Simpson PM. Last observation carried forward versus mixed models in the analysis of psychiatric clinical trials. Am J Psychiatry. 2009;166:639-41.
52. Singer JD, Willett JB. Applied longitudinal data analysis: Methods for studying change and event occurrence. New York: Oxford University Press; 2003.
53. Mennin DS, Fresco DM, Heimberg RG, Schneier FR, Davies SO, Liebowitz MR. Screening for social anxiety disorder in the clinical setting: using the Liebowitz Social Anxiety Scale. J Anxiety Disord. 2002;16:661-73.
54. Ballenger JC, Davidson JR, Lecrubier Y, et al. Consensus statement on social anxiety disorder from the International Consensus Group on Depression and Anxiety. J Clin Psychiatry. 1998;59 Suppl 17:54-60.
55. Fournier JC, DeRubeis RJ, Shelton RC, Hollon SD, Amsterdam JD, Gallop R. Prediction of response to medication and cognitive therapy in the treatment of moderate to severe depression. J Consult Clin Psychol. 2009;77:775-87.
56. Smits JA, Hofmann SG, Rosenfield D, et al. D-Cycloserine augmentation of cognitive behavioral therapy of social anxiety disorder: Prognostic and prescriptive variables. Under Review.
57. Enders CK. Missing not at random models for latent growth curve analyses. Psychol Methods. 2011;16:1-16.
58. Snijders TAB, Bosker RJ. Standard errors and sample sizes for two-level research. J Educ Stat. 1993; 18:237-59.
