# Supplementary material for: Dose Timing of D-Cycloserine to Augment Exposure Therapy for Social Anxiety Disorder: A Randomized Clinical Trial
Source: JAMA Netw Open. 2020 Jun 4;3(6):e206777. doi: 10.1001/jamanetworkopen.2020.6777 (PMC7273198; doi:10.1001/jamanetworkopen.2020.6777)
Supplement: Supplement 2. — Data Sharing Statement [file jamanetwopen-3-e206777-s002.pdf]

Data Sharing Statement

Smits. Dose Timing of D-Cycloserine to Augment Exposure Therapy for Social Anxiety Disorder. *JAMA Netw Open*. Published June 04, 2020. 10.1001/jamanetworkopen.2020.6777

Data
Data available: No

Additional Information
Explanation for why data not available: No IRB approval.
